# Diversification of Cellulose Synthase (*CESA*) Genes in Mosses Suggests Both Ancient and Recent Gene duplications

**DOI:** 10.1007/s00239-026-10342-3

**Published:** 2026-09-16

**Authors:** Alyssa Hartmann, Corinna Breusing, Alexandra Walling, Kerrina Whelan, Alison Roberts, Rachel S. Schwartz

**Affiliations:** Department of Biological Sciences, University of Rhode Island, Kingston, RI 02881 USA

**Keywords:** *Physcomitrium patens*, Gene family evolution, Paralogs

## Abstract

Cellulose is an important polysaccharide that constitutes all plant cell walls, giving them strength and stability. The plant cellulose synthase (*CESA*) gene family, which encodes the catalytic subunits of cellulose synthesis complexes (CSCs), has diversified independently in several plant lineages, providing an interesting model for understanding selection for gene duplication. Here we quantified the presence of *CESA* genes across mosses to understand how the process of gene family diversification occurred in this group and how it parallels diversification in other groups. We first examined the *CESA* gene family in eight species of mosses across seven families for which whole genome assemblies were available. We then identified *CESA* genes from additional species, for which only short-read sequence data was available, by using BLAST searches and targeted gene assemblies. We validated this approach by comparing the assembled paralogs from the short-read data to the genes identified from whole genome assemblies in the eight reference species. This approach allowed us to identify paralogs directly from short-read data and greatly expand our sample set. Results from the combined empirical data support the hypothesis that *CESA* genes diversified within the moss lineage at least as early as the mesozoic period, during or possibly even prior to the onset of moss diversification, but also continue to diversify within modern species. In addition, we found evidence for purifying selection as the dominant force shaping these genes and observed that different lineages experienced different levels of evolutionary constraint. Lastly, our approach to assemble paralogs has the potential to allow researchers to improve analyses of gene duplication events.

**Supplementary Information:**

The online version contains supplementary material available at https://doi.org/10.1007/s00239-026-10342-3.

## Introduction

Gene duplication is a key process in genome evolution that generates new genes with new functions. Following duplication, gene copies may diverge in function through the accumulation of mutations, followed by selection for either novel function (neofunctionalization) or specialization of multiple gene copies (subfunctionalization). Often, these duplicated genes that descend from a common ancestor gene can be seen as gene families due both to regions of sequence similarity and similar function (Gu 2001). Interestingly, gene duplication events are not distributed uniformly across organisms or genes, suggesting that both certain lineages and certain genes are more prone to maintaining duplications. When similar gene duplication events occur repeatedly it may suggest particular selective pressures and mechanisms by which organisms adapt to challenges.

The *CELLULOSE SYNTHASE* (*CESA*) gene family is a set of genes that shows repeated duplications across plants, but the timing and extent of duplication events is not completely known, especially in mosses, whose repeated duplications are thought to have occurred separately and in parallel to seed plants (Roberts and Bushoven 2006). The *CESA* gene family encodes the catalytic subunits of cellulose synthesis complexes (CSCs), which assemble cellulose microfibrils, an important component of plant cell walls (McFarlane et al. 2014). Plant cell walls are classified as either primary cell walls, which regulate cell growth, or secondary cell walls, which confer additional support (Keegstra 2010), and *CESA* gene family members are specialized for either primary or secondary cell wall biosynthesis in both vascular plants (McFarlane et al. 2014) and in mosses (Norris et al. 2017). The *CESAs* are members of a green plant-specific superfamily that includes several families of CESA-like (CSL) proteins, some of which participate in the synthesis of other cell wall polysaccharides (Little et al. 2018; Mutwil et al. 2008). The CESA proteins also share homology and enzymatic function with bacterial cellulose synthases (Morgan et al. 2013). The lack of knowledge of duplications in this gene family across mosses limits our understanding of how duplication events have contributed to structural and functional adaptations, and our understanding of the repeatability of duplication and specialization.

True *CESA*s have been identified in *Mesotaenium cladariorum* (Zygnematales) (Roberts et al. 2002), which represents the closest sister clade to land plants, among other charophycean green algae (Feng et al. 2024; Yin et al. 2014). The rosette CSCs of land plants, which have a 6-fold symmetry, are also present in members of the Charales and Zygnematales (green algae) (Tsekos 1999). CESAs have also been identified in the genome of the earlier diverging charophycean green alga *Klebsormidium nitens*, although the CSC type is unknown. The genome of the alga *Chlorokybus atmophyticus* (Chlorokybales) includes *CESA/CSLD*-like homologs, whereas no *CESAs* or *CSLDs* were identified in the genome of the alga *Mesostigma viride* (Mesostigmales) (Wang et al. 2020). This indicates that true rosette-forming CESAs evolved within the streptophyte (charophycean green algae/land plant) lineage after it diverged from chlorophycean green algae.

The *CESA* gene family appears to have diversified independently from a single common ancestor gene in seed plants, lycophytes, and bryophytes (i.e., mosses, liverworts and hornworts). Members of the six seed plant *CESA* classes are not found in spikemosses (Harholt et al. 2012) or true mosses (Roberts and Bushoven 2006). In *Arabidopsis thaliana*, each of the six clades contributes one of three distinct CESA isomers to the hetero-oligomeric CSCs that synthesize primary or secondary cell walls. The six *CESA* clades and corresponding functional classes are conserved in seed plants (McFarlane et al. 2014), but only partially conserved in ferns (Ali et al. 2025; Yin et al. 2014), indicating that rosette CSCs became hetero-oligomeric within the euphyllophyte (fern/seed plant) lineage, possibly starting before the divergence of ferns (Haigler and Roberts 2019).

Although its CESA family diversified independently of the seed plant CESA families, the moss *Physcomitrium patens* also has hetero-oligomeric CSCs that are specialized for primary and secondary cell wall biosynthesis (Norris et al. 2017), indicating that hetero-oligomerization, as well as functional specialization for primary and secondary cell wall deposition, evolved independently in the bryophyte and euphyllophyte plant lineages (Haigler and Roberts 2019). The *P. patens* CESA proteins form two clades that correspond to functional classes defined by genetic complementation (Li et al. 2022; Norris et al. 2017; Scavuzzo-Duggan et al. 2015). Class A consists of CESA3, CESA5 and CESA8, and class B includes CESA1, CESA4, CESA6, CESA7 and CESA10. Whereas the class B CESAs appear to be fully interchangeable (Scavuzzo-Duggan et al. 2015), additional functional subdivision is seen in class A (Li et al. 2022). While CESA3 and CESA8 function only in hetero-oligomeric CSCs with members of class B to produce secondary cell wall cellulose (Norris et al. 2017), CESA5 functions in homo-oligomeric CSCs to produce primary cell wall cellulose (Li et al. 2022). This functional differentiation implies a distinct history of *CESA* gene duplication and functional specialization within the bryophyte lineage. However, it is unknown whether *CESA* paralogs that encode functionally distinct CESA proteins have orthologs in other bryophyte species or to what extent *CESA* genes have diversified within specific bryophyte lineages.

Here we present a phylogenetic analysis of *CESA* genes from across the diversity of bryophytes (mosses, hornworts, and liverworts), with particular focus on mosses. Studying the divergence of the genes in a gene family across multiple related species can shed light on the gene duplication and specialization process (De Bie et al. 2006). We first obtained *CESA* genes from high-quality whole genome assemblies of eight moss species, three hornworts (*Anthoceros* spp.) and one liverwort (*Marchantia polymorpha*) as well as the charophyte *Penium margaritaceum* (Zygnematales), which represents the sister clade to bryophytes. However, because reference genomes have only been available for these few bryophyte species, to obtain paralogs from additional species we examined whether we could assemble individual genes directly from short-read sequence data using BLAST searches and targeted gene assemblies. We validated our approach by comparing the genes assembled from short-read data to those extracted from the whole genome assemblies for the same species. We were then able to use this approach to obtain paralogs for additional moss species. The large phylogeny presented here provides detailed information on the evolution of the CESA gene family across the diversity of mosses.

## Materials and Methods

### Data from Whole-Genome Assemblies

CESA sequences from whole genome assemblies (Bowman et al. 2017; Carey et al. 2021; Hu et al. 2023; Jiao et al. 2020; Lang et al. 2018; Weston et al. 2018; Yu et al. 2020, 2022; Zhang et al. 2020) were identified by pBLAST with the PpCESA5 protein sequence (Pp3c2_13330V3.1) as the query on species-specific genomes (Supplementary Table 1). The coding sequences were retrieved from gene models corresponding to the pBLAST hits. Each BLAST search returned sequences corresponding to CESAs and also CELLULOSE SYNTHASE-LIKE D (CLSD) protein sequences, which were distinguished from CESAs by the DAYNA/SHEE consensus sequence within the Plant Conserved region.

## CESA Gene Alignment and Phylogenetic Inference

We aligned nucleotide sequences based on their corresponding amino acid translations using TranslatorX (Abascal et al. 2010). Translated alignments were checked by eye to ensure no major gaps or errors. We estimated the phylogeny for the full nucleotide alignment using RAxML 8.2.12 (Stamatakis 2014) with a GTRGAMMA model with 100 rapid bootstraps.

## Gene Assemblies

To determine whether we could identify CESA genes from additional species directly from genomic or transcriptomic short-read sequences, we de novo assembled CESA genes from each species for which we had previously identified genes from whole genomes. Raw Illumina sequence reads (Supplementary Table 2) were downloaded from the Sequence Read Archive or the China National GeneBank Database and quality-trimmed with fastp (Chen et al. 2018) and trimmomatic (Bolger et al. 2014). Reads were then mapped against the human and PhiX genomes with bowtie2 (Langmead and Salzberg 2012) to remove common sequence contaminants. The final read set for each sample was mapped against all reference CESA gene sequences that were recovered from the previous BLAST searches, and matching reads were assembled with SPAdes (Bankevich et al. 2012) and/or Geneious Prime (https://www.geneious.com). The assembly resulting in the longest contigs was used for further analysis. Contigs that were at least 1,000 bp in length were aligned to the reference CESA gene sequences with MAFFT (Katoh et al. 2002) in Geneious Prime, and only contigs that showed sufficient similarity to these genes were retained. Contigs were then trimmed to the coding start and end of the CESA gene region to produce the final set of de novo assembled CESA genes. In case the full gene could not be recovered, poorly aligned intron regions at the beginning and end of the sequence were removed. Any contig with less than 1000 bp of alignable and translatable exonic region was excluded from further analysis.

## Phylogeny Inference

Phylogenies were inferred from the de novo assembled CESA gene sequences. Because some species had only RNAseq data, and introns were challenging to identify and align in some cases, only exons were included in the final alignment. Introns were removed based on alignment with the sequences extracted from whole genomes. Gene sequences were codon-aligned with the Translation Align tool in Geneious Prime using MAFFT as aligner and checked by eye. Any poorly aligned regions at the start and end of incomplete CESA contigs that resulted in large gaps were trimmed. Phylogenies were reconstructed with the GTRGAMMA model in RAxML (Stamatakis 2014) using automated bootstrapping (autoMRE) and visualized with the ggtree package in R (Yu et al. 2017).

## Gene Assemblies from Additional Data

The approach described above was then applied to 116 additional species of bryophytes to obtain CESA gene assemblies (Supplementary Table 2). Phylogenies were constructed as above. To assess the impact of partial sequences on phylogenetic reconstruction we further calculated the CESA tree including only nearly complete sequences, which we defined as sequences with at least 70% of the longest full-length CESA sequence.

### Selection Patterns in CESA Coding Sequences

To better understand the role of evolutionary forces in the diversification of the CESA gene family, we assessed selection patterns using CESA coding sequences from species with whole-genome assemblies. We implemented site and branch models in the CODEML program of PAML 4.9 (Yang 2007) to estimate dN/dS ratios. The dN/dS ratio (ω) compares the rate of nonsynonymous substitutions (dN) to the rate of synonymous substitutions (dS). A dN/dS ratio greater than 1 suggests positive selection, whereas a ratio less than 1 indicates purifying selection. Analyses followed Álvarez-Carretero et al. (2023). Sequences were codon-aligned and the resulting alignment was manually inspected in AliView (Larsson 2014). Stop codons and regions predominantly composed of gaps were removed prior to analysis. Because the selection analyses were restricted to sequences obtained from whole-genome assemblies, the larger dataset containing partial gene assemblies was not included in these analyses. The trimmed alignment was converted from FASTA to PHYLIP format with a custom R script for input to CODEML.

To detect selection across codon sites, we compared standard site-model pairs that do and do not allow for positively selected sites: M1a (Nearly Neutral) vs. M2a (Positive Selection) and M7 (beta) vs. M8 (beta + ω). For each model, a control file specified the input alignment, phylogenetic tree, and parameters. Log-likelihoods (lnL) were extracted from CODEML outputs and used for likelihood ratio tests (LRTs) to assess model fit; LRT calculations were automated in R.

To explore how selection has operated across CESA clades and assess variation among lineages, we applied branch models in CODEML to estimate lineage-specific dN/dS ratios. We compared a one-ratio model (model = 0, NSsites = 0; single ω across all branches), a free-ratio model (model = 1, NSsites = 0; separate ω for each branch), and two-ratio models (model = 2, NSsites = 0; separate ω for a designated foreground branch vs. background). In all branch-model fits, ω was estimated (fix_omega = 0). LRTs used the one-ratio model as the null for both free- and two-ratio analyses. Degrees of freedom were the difference in the number of free parameters between models; statistical significance was evaluated with a χ² distribution. For branch-specific two-ratio tests, raw p-values were adjusted for multiple comparisons using false discovery rate (FDR) control.

To test for episodic, lineage-specific positive selection acting on a subset of codons, we used the branch-site Model A in CODEML. For each designated foreground branch (tagged “#1” in the tree), we fit an alternative model that allows a site class with ω > 1 on the foreground (model = 2, NSsites = 2; fix_omega = 0) and a matched null model that fixes that class at ω = 1 (model = 2, NSsites = 2; fix_omega = 1, omega = 1). Models were compared by LRT with df = 1, and p-values were FDR-adjusted across foregrounds. When the alternative model was significant, we examined Bayes Empirical Bayes (BEB) posteriors and recorded sites with posterior ≥ 0.95 as candidates for positive selection on the foreground branch.

## Results

### Gene Phylogeny from Whole-Genome Data

**Fig. 1:**
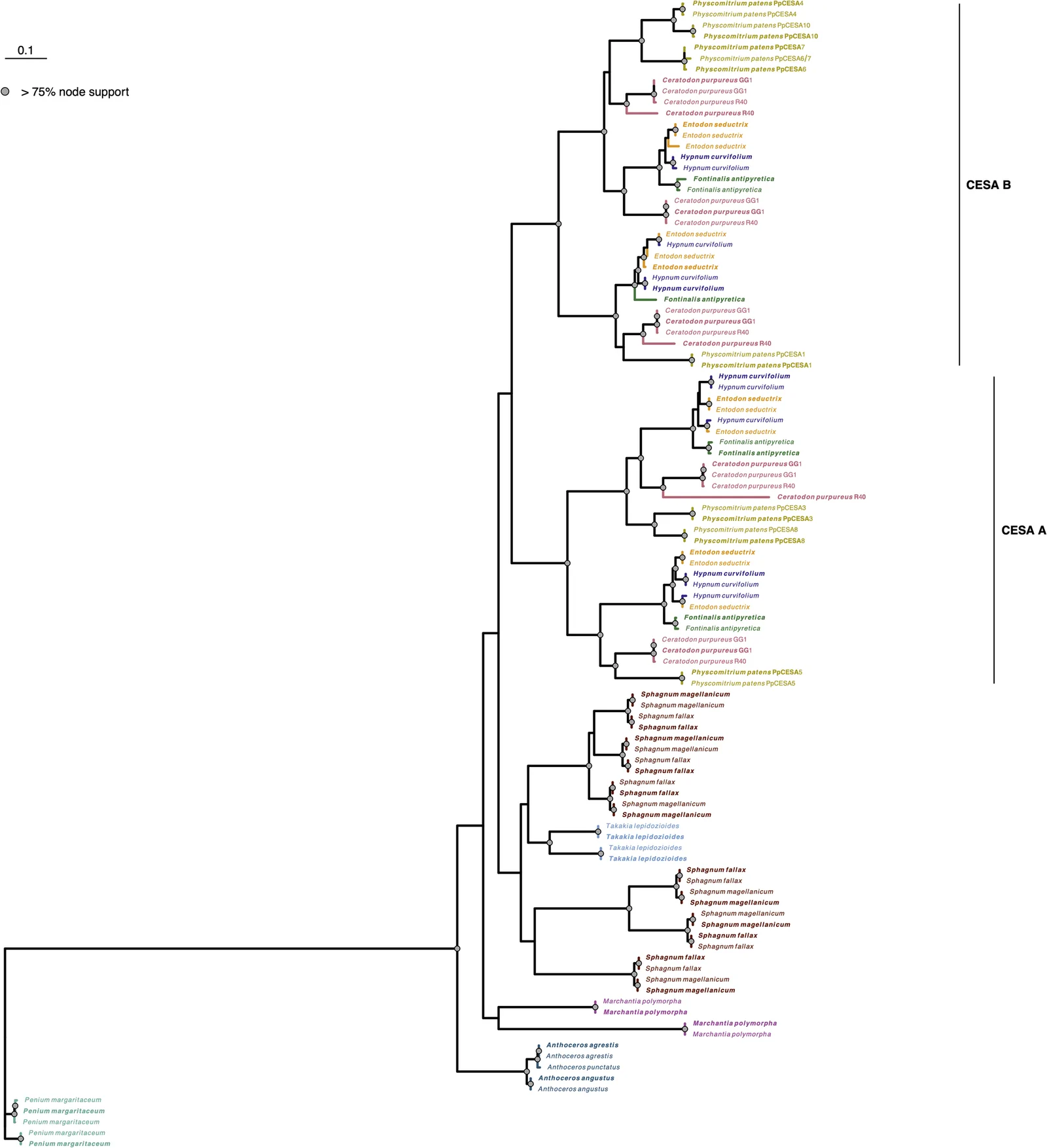
The phylogeny of CESA genes from eight species of mosses (colors) shows the validation of the assembly of genes directly from short-read data. Gene sequences in bold were obtained using BLAST searches on whole-genome assemblies. Plain-text sequences were obtained by mapping short-read sequences against reference CESA gene sequences and assembling the mapped reads. The genes obtained from the two approaches were nearly identical, supporting the identification of additional CESA sequences from unassembled short-read data. The phylogeny also shows an early split between the known A and B CESA classes (Scavuzzo-Duggan et al. 2018), as well as multiple genes within each class for most species. The charophyte alga *Penium margaritaceum* (Zygnematales) was used to root the tree, while a liverwort (*Marchantia polymorpha*) and three hornworts (*Anthoceros punctatus*,* A. agrestis*,* A. angustus*) were used to clarify the timing of diversification in CESA genes. The phylogeny was estimated in RAxML 8.2.1 with support values estimated using automated bootstrapping (autoMRE). Nodes with at least 75% bootstrap support are shown with grey circles. Established gene numbers are given for the genes in the model species *Physcomitrium patens*

The phylogeny for *CESA* genes in mosses based on sequences extracted from whole-genome assemblies recapitulates the two major well-supported clades of *CESA* genes known from *P. patens*: class A, a group containing *P. patens* genes *CESA3*,* CESA5*, and *CESA8*, and class B, containing *P. patens* genes *CESA1*,* CESA4*,* CESA6*,* CESA7*, and *CESA10* (Fig. 1). The last four are all more closely related to each other than to any other *P. patens* gene or sequence from another species. One ortholog for this set of genes is present in the other four members of Bryopsida, *Entodon seductrix*,* Hypnum curvifolium*,* Fontinalis antipyretica*, and *Ceratodon purpureus*, while another paralog is present exclusively in *Ceratodon purpureus.* All four of these Bryopsida species also contain an ortholog for *PpCESA1*. In Clade A, *PpCESA3* and *PpCESA8* are also more closely related to each other than to any other *P. patens* gene or sequence from another species. One ortholog for this pair of genes, as well as for *PpCESA5*, is present in the other four members of Bryopsida. Notably, each of the three hornwort species (*Anthoceros* spp.) has a single CESA gene that together form a sister clade to the moss and liverwort CESAs. Similarly, the two genes from the liverwort *Marchantia polymorpha* are closely related and not part of the moss CESAs.

### Targeted Gene Assemblies and Comparison to Whole Genome Data

De novo assemblies of Illumina reads identified as CESA resulted in 1–8 final genes per sample (Table 1). Assemblies were similar for Geneious and SPAdes; however, Geneious resulted in longer assemblies for all species except for *Hypnum curvifolium* (Table 1). In most cases, the number of sequences reflected the number of genes obtained through BLAST searches, although some notable exceptions existed. For *Fontinalis antipyretica* only two of the expected four gene sequences could be fully assembled, while a third sequence was partially recovered. Similarly, for *P. patens* full-length sequences could only be obtained for five of the eight expected genes, while PpCESA 4 and 10 were partially assembled. PpCESA 6 and 7 were assembled as a single gene; however this is not surprising as they are almost identical, including the introns, only differing by three amino acids. These two similar genes were only finally assembled correctly in the newest telomere-telomere genome based on long-read sequencing (Bi et al. 2024), and were previously only known to be distinct genes because they had been independently amplified and sequenced (Wise et al. 2011). In contrast, for *Entodon seductrix* and *Hypnum curvifolium* more paralogs were recovered than were obtained via BLAST searches. Additionally, in all cases, the de novo assemblies produced multiple smaller fragments that had similarity to CESA-like genes based on BLAST analyses. These fragments could not be assembled into larger contigs even when read depth was high. Information on the number and length of recovered genes for each species is provided in Supplementary Table 3.

Because some sequences were only partially recovered, and the estimate of the phylogeny depends on which portion of the gene is recovered, we examined the alignments for variation among samples. This allowed us to ensure that there appeared to be sufficient information to estimate the phylogeny. The alignment is provided as Supplementary Fig. 1.

**Table 1:** Sequence reads used to de novo assemble CESA genes to validate the assembly approach and the number of genes assembled from each species

Species	SRA/CNGD accession	Data type	Assembler	# Genes
*Anthoceros agrestis*	ERR3580402, ERR771108–109	WGS	Geneious	1
*Anthoceros angustus*	SRR9662965	RNAseq	rnaSPAdes	1
*Anthoceros punctatus*	SRR1278954	WGS	Geneious	1
*Ceratodon purpureus* GG1	SRR1592642–643, SRR3928188–192	WGS	Geneious	5
*Ceratodon purpureus* R40	SRR4125803–804	WGS	Geneious	5
*Entodon seductrix*	CL100124498	WGS	Geneious	8
*Fontinalis antipyretica*	ERR2040966	RNAseq	rnaSPAdes	3
*Hypnum curvifolium*	CL100092064	WGS	SPAdes	7
*Marchantia polymorpha*	SRR396657–658	WGS	Geneious	2
*Penium margaritaceum*	SRR10442432	WGS	Geneious	3
*Physcomitrium patens*	SRR3099021	WGS	Geneious	7
*Sphagnum fallax*	SRR4136564, SRR4136566	WGS	Geneious	6
*Sphagnum magellanicum*	SRR4996476–477	WGS	Geneious	6
*Takakia lepidozioides*	SRR17573916–920	WGS	Geneious	2

The phylogeny combining the genes extracted from the whole-genome assemblies and the genes assembled from Illumina reads shows each assembled sequence paired with a sequence from the whole genome. The additional paralogs found in *Entodon seductrix* and *Hypnum curvifolium* appear to be recent duplications based on the phylogeny, as they are each sister to existing genes (Fig. 1).

### Phylogeny from 130 Species Using Whole Genome and De Novo Assembled Sequences

De novo assemblies of Illumina reads identified as CESA resulted in 1–14 final genes per sample (Supplementary Table 3). Assemblies ranged in size from 1017 to 3414 bp, with a median of 3273 bp. 131 genes (out of 536) were considered “partial sequences” as they had a length of less than 70% of the longest complete gene of 3414 bp. Information on the length of each gene is provided in Supplementary Table 3. The alignment is provided as Supplementary Fig. 2.

**Fig. 2:**
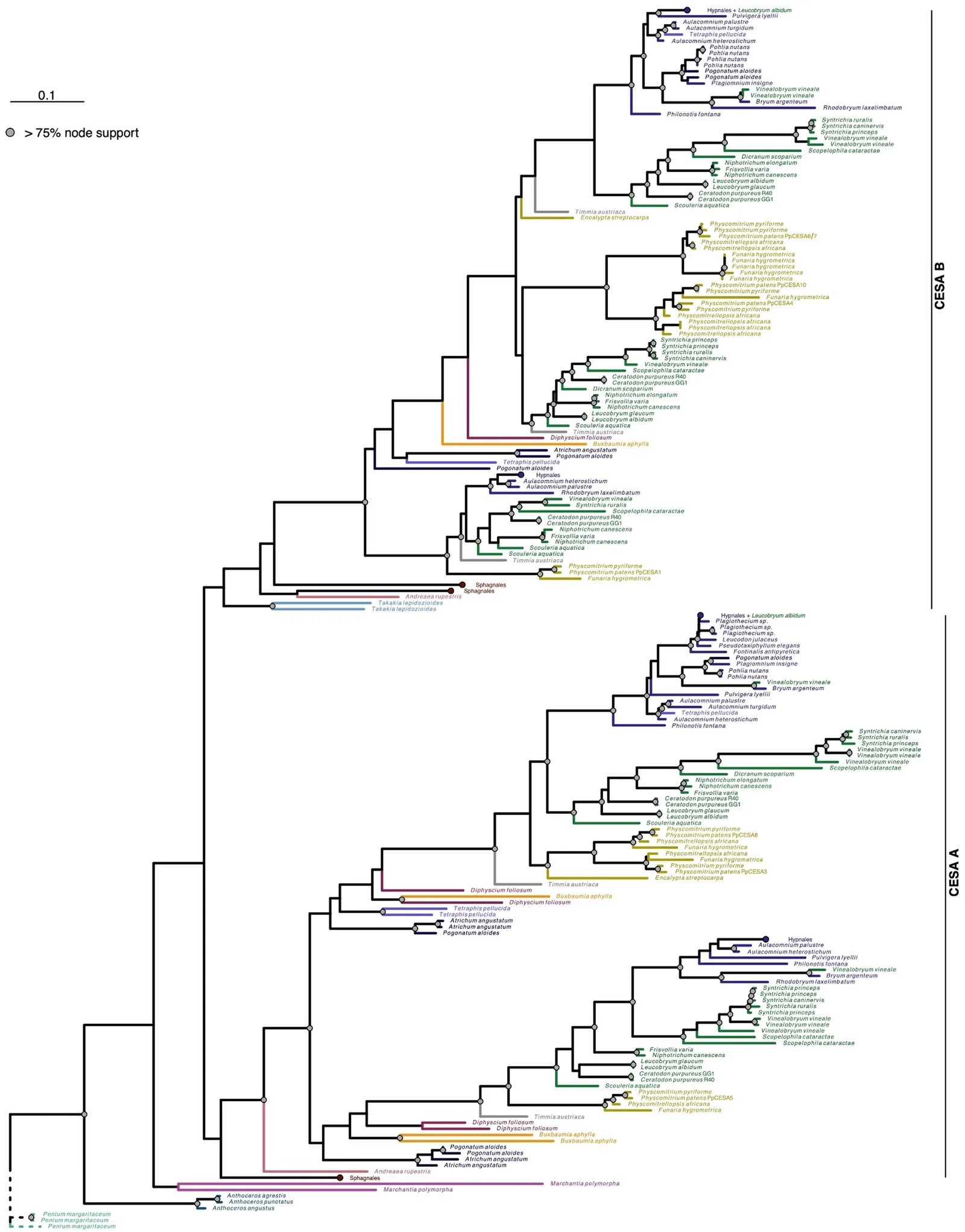
The phylogeny of CESA genes from 130 species of mosses. Sequences were obtained by mapping short-read sequences against reference CESA gene sequences and assembling de novo. Some supported clades are collapsed to allow improved viewing (the full phylogeny is available as Supplementary Fig. 3). These are marked with a diamond and include Sphagnales and Hypnales. Nodes with bootstrap > 75 are marked with a grey circle

The phylogeny including all 130 species retains the division between the class A and class B *CESA*s (Fig. 2). *CESA* sequences from *Takakia lepidozioides* are more closely related to each other than to genes in any other species and they appear to be in the B clade, although this is not strongly supported. Genes from Sphagnales are assigned to both A and B clades, although bootstrap support assigning the Sphagnales genes to these clades is relatively low (Fig. 2). Within each clade (A and B) Sphagnales has three strongly supported genes diverging before the divergence of species within the family and unrelated to the split within other Bryophyta (Supplementary Fig. 1).

Class A contains two major clades: one with orthologs for *P. patens CESA5* and the other with orthologs for *CESA3 and 8.* Species in Pseudoditrichales, Hypnales, Orthotrichales, Scouleriales, Pottiales, Polytrichales, Tetraphidales, Hypnales, and Diphysciales all have at least one CESA gene that is orthologous to *CESA3* and *CESA8* in *P. patens*, as well as *CESA5*. Thus, the CESA genes that are labeled 3 and 8 in *P. patens* apparently split from *CESA5* prior to diversification into these groups. In contrast, Takakiales do not have orthologs for both genes. Thus, this gene duplication occurred after the split between Takakiales and the families listed previously.

Within the genes that are orthologous to *CESA3* and *CESA8* there are multiple independent duplications. *CESA3* and *CESA8* diverged prior to the diversification of Funariales, as both genes are found in each species of this family. There are also recent duplications in *Tetraphis*,* Pogonatum*,* Pohlia*, and *Vineolobryum*, among others. Within the genes that are orthologous to *CESA5,* duplications are observed for multiple species within Hypnales and Pottiales, *Timmia austriaca*, *Diphyscium foliosum*, *Buxbaumia aphylla*, *Pogonatum aloides*, and *Atrichum angustatum*.

Class B contains two major clades: one with orthologs for *P. patens CESA1* and the other with orthologs for *CESA10*,* 6/7*, and *4.* Almost all families have a single ortholog for *CESA1.* In contrast there is extensive diversification in the latter (Fig. 2 and Supplementary Fig. 3). Recent diversification within the Funariales, known from the four *P. patens* genes, is also present in other species. Additional recent diversification is also present in *Pohlia*,* Syntrichia*,* Vinealobryum*, and others.

When including only nearly complete sequences in the phylogenetic reconstruction, the tree topology is generally preserved, supporting the larger splits into CESA A and B clades and observed group relationships within these clades. One notable exception is the placement of one of the classes of Sphagnales CESA sequences as sister to *Takakia* and clade B rather than the CESA A clade, though statistical support for this placement is low (Supplementary Fig. 4).

### Selection Patterns in Conserved CESA Regions

The likelihood ratio tests of site models rejected models that were consistent with positive selection in favor of models of neutral or purifying selection (Table 2). No codon sites showed statistically significant evidence of ω > 1 under Bayes Empirical Bayes (BEB) analysis, further supporting the conclusion that these genes are functionally constrained.

**Table 2:**
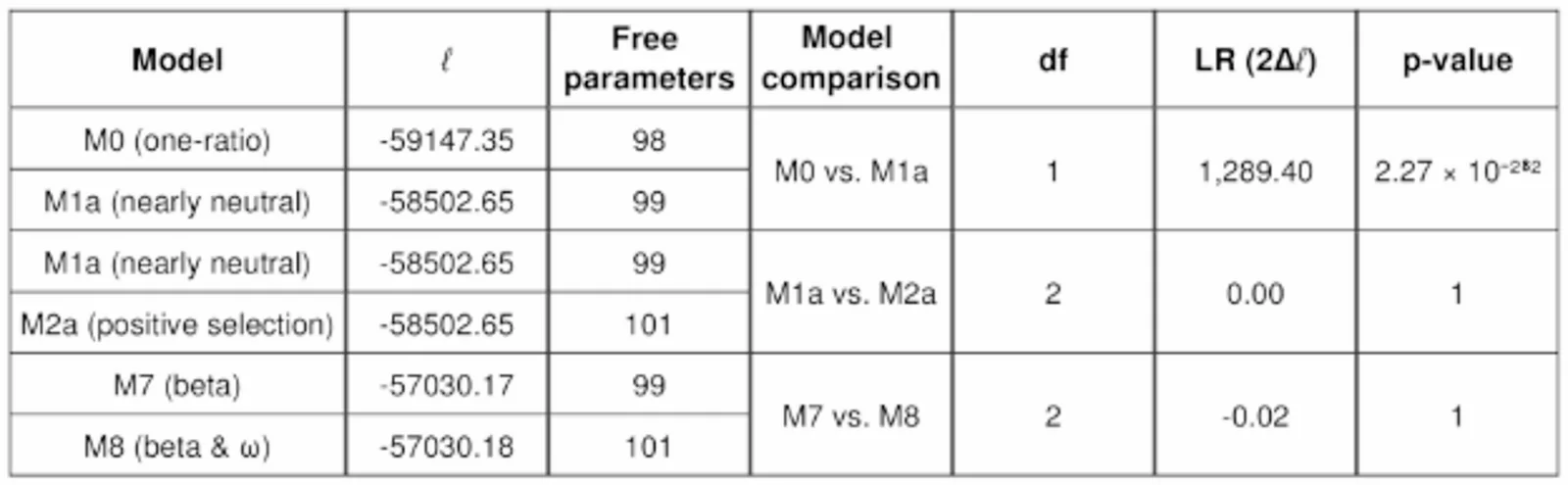
Summary of site model comparisons and likelihood ratio test results for detecting selection using CODEML

**Fig. 3:**
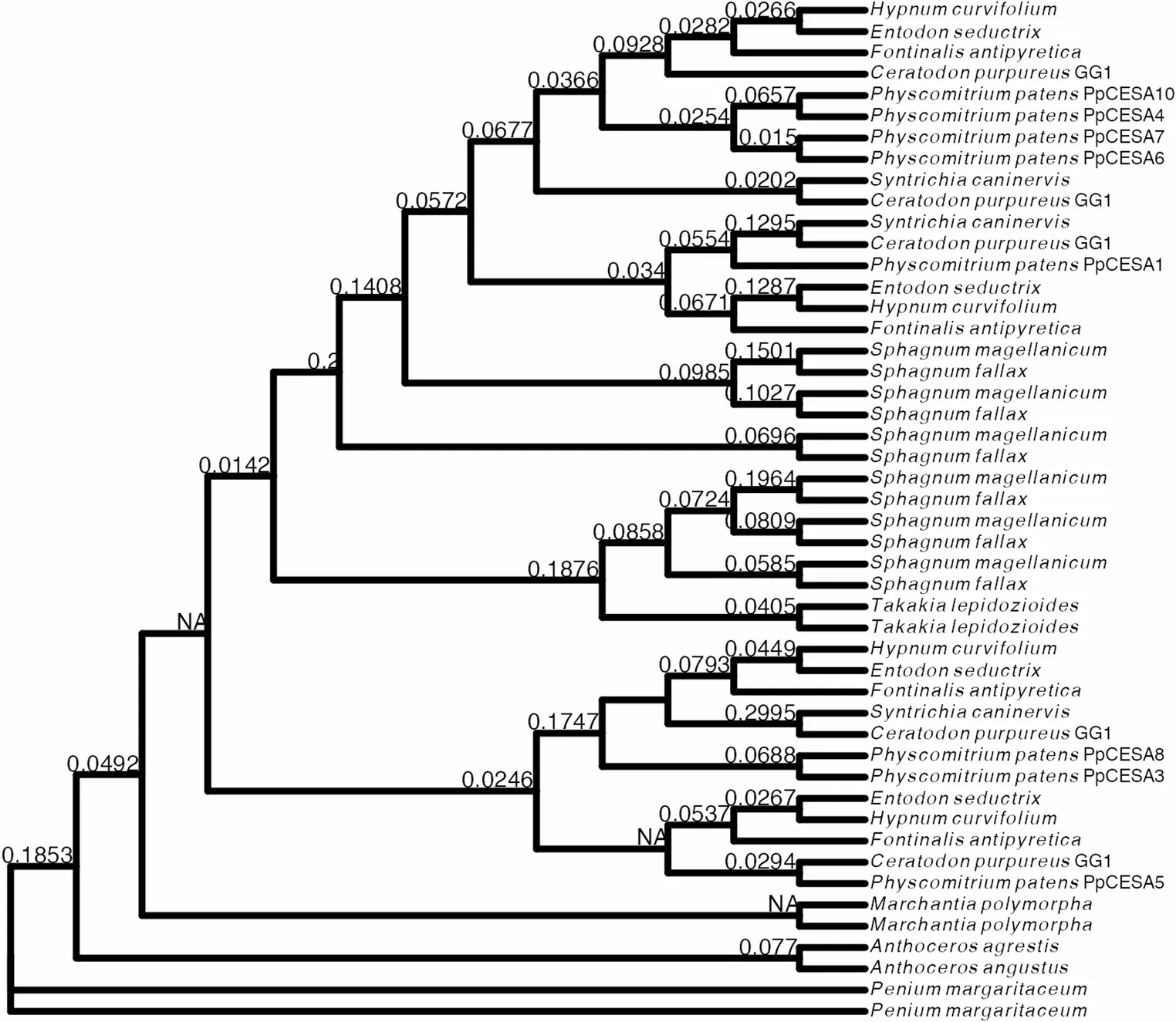
Phylogeny of CESA genes showing branch-specific dN/dS (ω) values estimated with the free-ratio branch model in CODEML, which assigns an independent ω to each branch of the tree. CODEML analyses were run under runmode = 0 (user tree), seqtype = 1 (codons), and model = 1 (free-ratio), providing maximum-likelihood estimates of dN, dS, and ω for each lineage. Estimates ranged from 0.0142 to 0.2995, with all values below 1, consistent with purifying selection as the dominant evolutionary force shaping these genes. Evidence for site-specific positive selection was assessed separately using CODEML site models (Table 2)

Across all tested branches, ω values were consistently below 1, indicating purifying selection is the dominant force shaping these genes. The free-ratio model (model = 1) provided a significantly better fit to the data than the one-ratio model (*p* < 0.05), suggesting that different lineages experience varying levels of evolutionary constraint rather than a uniform ω across the tree. The two-ratio branch model identified 15 branches for which allowing a branch-specific ω significantly improved model fit relative to the one-ratio model after false discovery rate (FDR) correction, indicating heterogeneity in the strength of purifying selection across the phylogeny (Supplementary Table 4). Branch-specific dN/dS values are shown on the annotated phylogenetic tree in Fig. 3. Some branches exhibited high dN/dS values (ω > 3), likely due to low synonymous substitution rates (dS < 0.01) or alignment artifacts. We removed information for branches with dS < 0.01 or dS > 3, as such cases can artificially inflate ω, and dN/dS > 10 to eliminate inaccurate ω estimates.

Branch-site tests for episodic, lineage-specific positive selection identified three foreground branches (out of 48 tested) that were significant after FDR correction. For these branches, the alternative fits indicated a small class of sites under selection on the foreground (ω_2_ ~ 1.5) comprising approximately 1–4% of codons, and no individual sites reached BEB ≥ 0.95. All remaining foreground branches were not significant after FDR, indicating that while selective pressures vary among lineages (as shown by branch models), we detect limited evidence for episodic positive selection confined to specific branches and sites in conserved CESA regions (Supplementary Table 5).

All analysis scripts can be found at https://github.com/rachelss/CesA_Analysis.

## Discussion

Phylogenetic analysis of *CESA* genes indicates that orthologs of the *P. patens* class A and class B *CESA*s are represented in the genomes of members of the Bryopsida, as well as the earlier diverging Polytrichales, Andreales, and Sphagnales (although there was some uncertainty in the placement of the genes in the latter group). In contrast, we only identified CESA genes with a single unclear clade association in *Takakia lepidozioides*, representing the Takakiales. Thus, the clade A and clade B *CESA*s likely diverged before the Sphagnales diverged from the rest of the moss lineage about 351 mya (Hu et al. 2023), and we suggest that hetero-oligomeric CSCs composed of different isoforms could have evolved around the same time. The Takakiales are believed to be the earliest divergent moss lineage about 390 mya (Hu et al. 2023). Thus, the absence of clade-associated *CESA*s in the whole genome sequence of *Takakia lepidozioides* is consistent with a later divergence of class A and class B. However, the loss of a second clade of CESAs in this lineage cannot be ruled out.

In *P. patens*, class A (PpCESA3 and −8) and class B (PpCESA6 and −7) subunits form the CSCs that synthesize cellulose in the secondary cell walls of stereid cells in the leaf midribs (Li et al. 2019, 2022; Norris et al. 2017). The recent divergence of PpCESA3 and −8 is consistent with functional redundancy of these paralogs. PpCESA6 and −7 are also functionally redundant and nearly identical (Norris et al. 2017). The Takakiales lack midribs with thick secondary walls (Hu et al. 2023), which is consistent with an absence of orthologs for genes in clade B (*PpCESA5* and *PpCESA3/8*). Although the Sphagnales also lack midribs, they do have hyaline cells with secondary wall thickenings. Like the stereid cells of *P. patens* and xylem elements of vascular plants (Xu et al. 2014), the development of hyaline cells is regulated by NAC family transcription factors, suggesting that a secondary cell wall regulatory module may have been repurposed in different land plant lineages (Liu et al. 2024). Transcriptional regulation of CESAs by NAC transcription factors has been demonstrated only in *P. patens* (Xu et al. 2014) and vascular plants (Demura and Ye 2010). Information from mutation and gene expression studies may shed light on the regulation and functional specialization within the *Sphagnum* CESA families.

Although the CESA families diversified independently in mosses and seed plants, both lineages converged on hetero-oligomeric CSCs with isoforms specialized for primary and secondary wall deposition (Li et al. 2022; Norris et al. 2017). Our analysis shows that moss CESA genes are highly conserved, with branch-specific dN/dS ratios consistently below 1 and no strong evidence of site-specific positive selection. These relatively low ω values indicate strong purifying selection, suggesting that critical catalytic and structural domains of the proteins have been maintained over time. Similar patterns have been observed in grasses, where most CESA/CSL clades show ω well below 1 (Schwerdt et al. 2015), and in woody species such as teak, where CESAs are also dominated by purifying selection with only a few codons showing signs of episodic positive selection (Balakrishnan et al. 2024). Consistent with this overall conservation, branch-site analyses yielded a limited signal confined to three lineages and did not pinpoint confidently supported codons, suggesting that any episodic selection has been sparse in the conserved regions examined.

Although ω values varied modestly among moss branches, the overall pattern suggests that CESA diversification was shaped more by regulatory changes and functional partitioning than by protein sequence evolution. This aligns with observations in other multimeric complexes, where duplicated subunits are retained under purifying selection while diverging in expression or regulation to achieve specialization (Finnigan et al. 2012). By contrast, some angiosperms show evidence of adaptive changes in cellulose synthase lineages. In grasses, dN/dS analyses identified codons under episodic positive selection in several CESA clades following duplication, consistent with adaptive diversification (Schwerdt et al. 2015). In cotton, however, CESAs appear to have evolved under purifying selection, with little evidence of amino acid–level adaptation despite their central role in fiber development (Zou et al. 2018).

The absence of widespread positive selection in moss CESAs may reflect their essential catalytic role in cellulose synthesis, where most amino acid substitutions are likely deleterious and removed by purifying selection. Diversification could therefore have occurred primarily through regulatory divergence or subfunctionalization after duplication, rather than through changes in protein sequence. It is also possible that the strong conservation of moss CESAs limits statistical power to detect rare episodic selection (Kosakovsky Pond and Frost 2005) or that mosses did not experience the ecological and developmental pressures that drove adaptive evolution in specific seed plant lineages. Overall, our findings indicate that moss CESAs diversified under strong purifying selection, with specialization emerging mainly through regulatory divergence rather than widespread protein-level adaptation.

### Gene Synteny

**Fig. 4:**
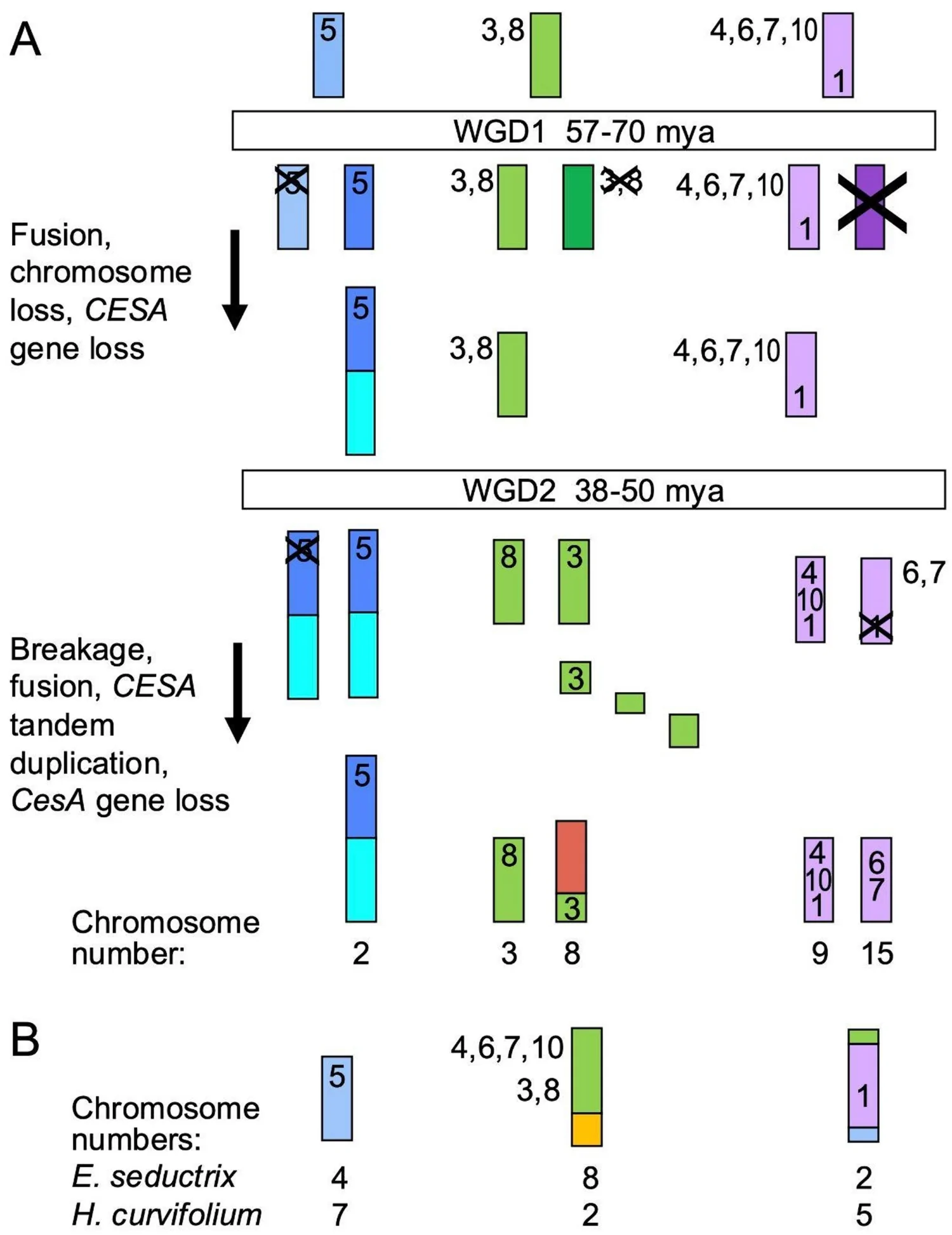
CESA synteny analysis in mosses. **A** Hypothesis for *PpCESA* gene evolution based on evolution of the *P. patens* karyotype through two whole-genome duplications (WDG) as illustrated and color-coded in Fig. 5 C in Lang et al. (2018). Duplication in WGD1 of the chromosome carrying the ancestor of *PpCESA5* (light blue) was followed by fusion of one duplicate (dark blue) with another chromosome (aqua) and loss of the paralog from the other duplicate (light blue). Duplication of the fused chromosome in WGD2 was followed by loss of the paralog from chromosome 1, resulting in a single remaining paralog, *PpCESA5*, residing on chromosome 2. Duplication in WGD1 of the chromosome carrying the ancestor of *PpCESA3* and *−8* (light green) was followed by loss of the paralog from one duplicate (dark green). WGD2 produced paralogs *PpCESA8* residing on chromosome 3 and *PpCESA3* residing on chromosome 8, which arose through breakage of the chromosome 3 duplicate and fusion of the fragment containing *PpCESA3* with another chromosome fragment (dark orange). Duplication of a third chromosome carrying the ancestor of *PpCESA1* and the common ancestor of *PpCESA4*, *−6*, *−7* and *−10* was followed by the loss of one duplicate chromosome. After WGD2, the *CESA1* paralog was lost from chromosome 15 and *CESA1* remained on chromosome 9. *CESA4* and *−10* arose from an early tandem duplication of the other *CESA* gene on chromosome 9 and *CESA6* and *−7*, which are nearly identical, arose from a more recent tandem duplication of its paralog on chromosome 15. **B** The *CESA*-containing chromosomes of *H. curvifolium* and *E. seductrix* are color-coded according to synteny with *P. patens* based on Figs. S3 and S4 in Yu et al. (2022). Orthologs of *PpCESA1* and *PpCESA3/8* are located on the corresponding *H. curvifolium* and *E. seductrix* chromosomes. Orthologs of *PpCESA4*/*6*/*7*/*10* are located in a different genomic context on the same chromosomes as the orthologs of *PpCESA3*/*8*

The recent divergence of some *P. patens* genes (*PpCESA*4, −6, −7, and −10; *PpCESA3* and *−8*) is consistent with evidence for a whole genome duplication (WGD) in the Funariales 38–50 mya, whereas the class A and class B clades predate an older WGD 57–70 mya that was shared by Funaridae and its sister clade, which includes Timmiidae, Dicranidae, and Bryidae (Gao et al. 2022; Rensing et al. 2007; Wang et al. 2022). We provide an illustration of the hypothesized chromosomal changes concomitant with CESA diversification (Fig. 4) consistent with our phylogenetic analysis. The eight *PpCESA*s reside on chromosomes descended from three of the seven chromosomes postulated to have existed before WGD1 (Fig. 4A). This supports the early divergence of the class A and class B *CESA*s, as well as divergence of *PpCESA5* and the common ancestor of *PpCESA3* and *−8*. In the most parsimonious scenario, WGD2 created *PpCESA3* and *−8* through duplication of the common ancestor of chromosomes 3 and 8, and the *PpCESA4*/*10* and *CESA6*/*7* common ancestors through duplication of the common ancestor of chromosomes 9 and 15. *PpCESA4* and *−10* appear to be a proximal duplicated pair with orthologs in *Physcomitrium pyriforme*, but not other Funariales genera, whereas the nearly identical *PpCESA6*/*7* tandem pair (Roberts and Bushoven 2006) together share one or more orthologs in other Funariales species, consistent with a more recent duplication compared to *PpCESA4* and *−10*. The *PpCESA1* duplicate from WGD2 was lost from chromosome 15, leaving a single copy that clusters with orthogs from other species. Following each WGD, one copy of *PpCESA5* was lost leaving a single copy (chromosome 2) that also clusters with orthologs from other species. Extensive synteny of *P. patens* with *H. curvifolium* and *E. seductrix* (Yu et al. 2022) shows that the genes identified as *PpCESA1*, *PpCESA3/8*, and *PpCESA5* orthologs in these species also share their genomic context. In contrast, the orthologs of *PpCESA4*/*6*/*7*/*10* are located in a different genomic context, likely due to translocation. There is also evidence for an earlier WGD shared by all mosses including Takakiales (Gao et al. 2022; Wang et al. 2022). If this event created the class A and class B *CESAs*, then class A may have been lost in the Takakiales. However, Gao et al. (2022) note that they cannot rule out the possibility that duplications attributed to a moss-wide WDG could have resulted from accumulation of individual gene duplications.

### Genome-Extracted Genes v. Gene Assemblies

De novo assembly of target genes directly from short reads allowed the extraction of additional information about recent gene duplication beyond searches of whole genomes, which typically require genome assembly, often using long-read data. We did observe cases where more genes were assembled from short-read data than were extracted from the whole-genome assembly, and vice versa. Assemblies are known to assemble closely related genes such that they appear to be one gene (Bailey et al. 2004), which may account for the additional genes we recovered in *Entodon seductrix* and *Hypnum curvifolium* that were not in the genome assemblies. The additional smaller CESA-like fragments produced in the de novo assembly process may simply represent noise; however, it is possible that additional closely related paralogs exist that were not correctly assembled in whole-genome assemblies. While we were also unable to assemble these fragments further, it is possible they warrant further exploration as recent gene duplications.

Conversely, the long-read data used to assemble whole genomes may be responsible for including all eight CESA genes in *P. patens* while we recovered only five full-length sequences and two partial assemblies. This result could be due to insufficient read depth for us to successfully assemble all sequences. Insufficient read depth or differential expression of *CESA* transcripts in RNASeq data might also explain why no or fewer-than-expected *CESA* genes were assembled in some other species. This observation is supported by the fact that potentially viable CESA fragments were observed in some species but were not included in downstream analyses due to their short lengths. This issue might require further attention in the future when additional sequence data for moss species become available. Full genome information will help to identify whether some of the short fragments recovered here represent additional functional *CESA* genes, pseudogenes, or parts of the same paralog. Alternatively, in some cases (such as the known closely related nearly identical *P. patens* genes) it may be challenging to assemble all genes regardless of read depth as genes are extremely similar due to a recent divergence. While this challenge does limit inference of the most recent divergences, it does not affect our larger conclusions here regarding repeated divergences across history for CESA genes in mosses. With the exception of the challenges of assembling all copies of recently diverged genes, the recovery of nearly identical sets of genes from our two approaches suggests both that the genome assemblies were successful in assembling nearly all copies of the genes, and that our approach using BLAST and targeted assembly also obtains nearly all genes.

There is some degree of mismatch between phylogenies for each gene and the species phylogeny for mosses. Given the lack of information in each *CESA* gene, differences between the species tree and the gene tree are not surprising. Thus, the phylogeny of the species should be inferred from larger datasets of sequences from across the genome rather than taken from this work (e.g. Hu et al. 2023). However, the species relationships are generally consistent with the phylogeny expected from family-level analyses (Wang et al. 2022) suggesting the patterns of duplication that we observe are reliable.

## Conclusions

The *CESA* phylogeny for mosses shows repeated duplications of *CESA* paralogs at different scales including within a single species, within groups of mosses prior to species divergence, and across mosses as a whole. This result is consistent with the observations of separate, repeated divergences within land plants (Harholt et al. 2012; Roberts and Bushoven 2006). These duplications may have occurred via any of various mechanisms, including whole genome duplication (alloploidy or autoploidy), proximal duplicated pairs, and tandem duplicated pairs. The retention of genes arising from repeated duplications at different time scales reflects the adaptive advantage of maintaining multiple *CESA* isoforms in different plant lineages. Thus, the evolution of *CESA* genes shows both the conservation of function to maintain the cellulose structure and the flexibility of the specific genes that code for protein components that make up the rosette CSCs.

Additionally, we were able to examine gene duplications and understand gene family evolution both by extracting genes from whole genome assemblies and assembling genes directly from short-read sequence data. This approach has the potential to be valuable in research on gene family evolution in cases where fewer well-assembled whole genomes are available. Furthermore, the ability to separate even closely related gene copies has significant potential for research in phylogenetics, where cryptic paralogs can alter estimates of species evolution (Brown and Thomson 2017).

## Supplementary Information

Below is the link to the electronic supplementary material.

Supplementary Material 1

Supplementary Material 2

Supplementary Material 3

Supplementary Material 4

Supplementary Material 5

## Data Availability

Data are available from the European Nucleotide Archive using the accession numbers listed in the paper. Whole genomes were obtained from Phytozome (https://phytozome-next.jgi.doe.gov/) and Bryogenomes (http://www.bryogenomes.org/). Alignments for the phylogenetic and selection analyses, as well as scripts to regenerate the alignments and repeat all analyses are found at https://github.com/rachelss/CesA_Analysis.
