# Supplementary material for: Diversification of Cellulose Synthase (CESA) Genes in Mosses Suggests Both Ancient and Recent Gene duplications
Source: J Mol Evol. 2026 Sep 16;94(5):907–21. doi: 10.1007/s00239-026-10342-3 (PMC13672439; doi:10.1007/s00239-026-10342-3)
Supplement: Supplementary file 1 [file 239_2026_10342_MOESM1_ESM.pdf]

NCBI Multiple Sequence Alignment Viewer, Version 1.26.0

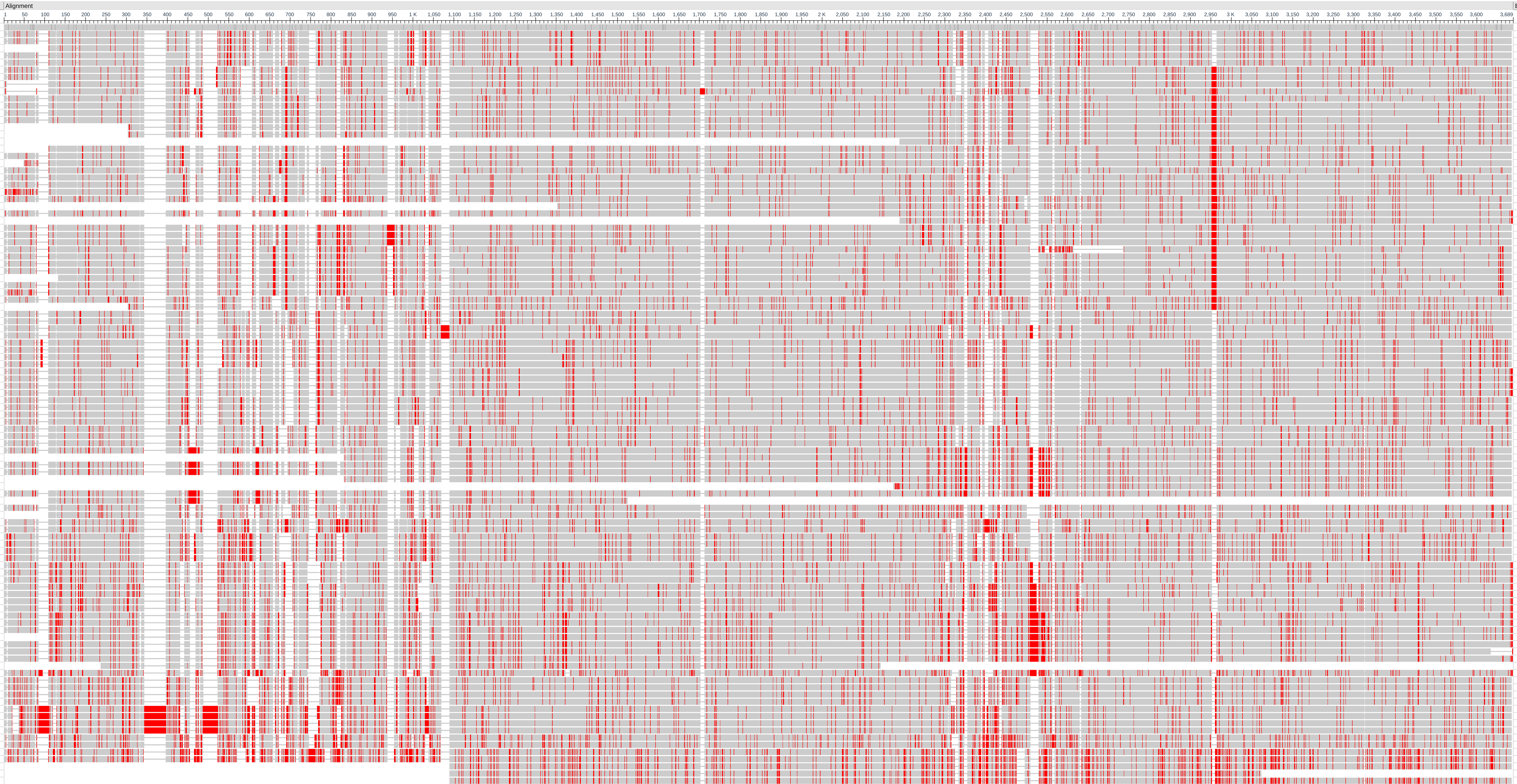

Sequence ID	Start	End	Identity	Coverage	Mismatches
consensus (+)	1	3,689	100.00	100.00	0
Anthoceros_agrestis (+)	1	3,303	82.76	89.54	254
Anthoagr.Sc2ySw. (+)	1	3,303	82.76	89.54	254
Anthoceros_puncta.. (+)	1	3,225	83.14	87.42	252
Anthoceros_angus.. (+)	1	3,303	83.03	89.54	244
Anthoang012329 (+)	1	3,303	83.03	89.54	244
Ceratodon_purpur.. (+)	1	3,294	83.33	89.29	224
CepurGG1.10G09.. (+)	1	3,294	83.33	89.29	224
Ceratodon_purpur.. (+)	1	3,222	81.54	87.34	218
CepurR40.g11450 (+)	1	3,249	80.97	88.07	266
Foant03732 (+)	1	3,309	84.34	89.70	202
Hypjg25231 (+)	1	3,309	84.74	89.70	187
Hypnum_curvifoli.. (+)	1	3,309	84.74	89.70	187
Entjg16709 (+)	1	3,309	84.77	89.70	186
Entodon_seductrix_6 (+)	1	3,042	84.62	82.46	181
Entodon_seductrix_2 (+)	1	3,045	84.24	82.54	197
Hypnum_curvifoli.. (+)	1	1,422	89.69	38.55	82
Ceratodon_purpur.. (+)	1	3,210	84.59	87.02	185
CepurGG1.7G024.. (+)	1	3,288	84.15	89.13	188
Ceratodon_purpur.. (+)	1	3,249	83.80	88.07	202
CepurR40.g14392 (+)	1	3,294	83.66	89.29	212
PpCESA6 (+)	1	3,291	83.93	89.21	199
PpCESA7 (+)	1	3,291	83.90	89.21	200
Physcomitrium_pa.. (+)	1	3,291	83.17	89.21	227
PpCESA10 (+)	1	3,288	81.89	89.13	271
Physcomitrium_pa.. (+)	1	2,238	89.92	60.67	142
PpCESA4 (+)	1	3,300	82.97	89.46	241
Physcomitrium_pa.. (+)	1	1,425	88.51	38.63	100
Ceratodon_purpur.. (+)	1	3,321	83.90	90.02	230
Ceratodon_purpur.. (+)	1	3,321	83.88	90.02	231
CepurGG1.1G272.. (+)	1	3,321	83.90	90.02	230
Foant02392 (+)	1	3,189	80.10	86.45	238
Entjg12779 (+)	1	3,318	84.85	89.94	192
Entodon_seductrix_5 (+)	1	3,318	84.85	89.94	192
Hypjg10340 (+)	1	3,318	84.80	89.94	194
Entodon_seductrix_1 (+)	1	3,222	85.30	87.34	192
Fontinalis_antipyr.. (+)	1	3,318	84.34	89.94	211
Hypnum_curvifoli.. (+)	1	3,318	84.31	89.94	212
PpCESA1 (+)	1	3,291	81.76	89.21	279
Physcomitrium_pa.. (+)	1	3,024	81.78	81.97	259
Tle4c03737 (+)	1	3,309	84.01	89.70	214
Takakia_lepidozioi.. (+)	1	3,309	84.01	89.70	214
Tle3c00983 (+)	1	3,318	83.88	89.94	228
Takakia_lepidozioi.. (+)	1	3,318	83.88	89.94	228
Sphfalx17G024100 (+)	1	3,273	82.52	88.72	233
Sphagnum_fallax_6 (+)	1	3,273	82.52	88.72	233
Sphmag17G023300 (+)	1	3,273	82.49	88.72	234
Sphagnum_magel.. (+)	1	3,273	82.49	88.72	234
Sphfalx19G037000 (+)	1	3,276	84.05	88.80	177
Sphagnum_fallax_3 (+)	1	3,276	84.05	88.80	177
Sphmag19G038600 (+)	1	3,276	84.00	88.80	179
Sphagnum_magel.. (+)	1	3,276	84.00	88.80	179
Sphfalx16G018300 (+)	1	3,285	83.63	89.05	204
Sphagnum_fallax_5 (+)	1	3,285	83.63	89.05	204
Sphmag16G006100 (+)	1	3,285	83.66	89.05	203
Sphagnum_magel.. (+)	1	3,285	83.66	89.05	203
Ceratodon_purpur.. (+)	1	3,261	83.01	88.40	203
Ceratodon_purpur.. (+)	1	3,261	82.84	88.40	209
CepurGG1.6G157.. (+)	1	3,261	83.01	88.40	203
Entjg6929 (+)	1	3,318	81.98	89.94	298
Entodon_seductrix_7 (+)	1	2,688	86.82	72.87	211
Hypjg27744 (+)	1	3,318	81.81	89.94	304
Hypnum_curvifoli.. (+)	1	3,318	81.81	89.94	304
Entodon_seductrix_4 (+)	1	2,688	86.54	72.87	219
Hypnum_curvifoli.. (+)	1	1,437	84.89	38.95	156
Foant15610 (+)	1	3,327	82.08	90.19	303
Fontinalis_antipyr.. (+)	1	1,167	73.73	31.63	123
PpCESA5 (+)	1	3,246	80.97	87.99	263
Physcomitrium_pa.. (+)	1	3,168	81.49	85.88	254
Mapoly0076s0014 (+)	1	3,327	82.00	90.19	306
Marchantia_polym.. (+)	1	3,327	82.00	90.19	306
Sphfalx13G080300 (+)	1	3,273	80.46	88.72	309
Sphagnum_fallax_4 (+)	1	3,273	80.46	88.72	309
Sphmag13G079500 (+)	1	3,288	80.89	89.13	308
Sphagnum_magel.. (+)	1	3,288	80.89	89.13	308
Ceratodon_purpur.. (+)	1	3,261	80.80	88.40	282
Ceratodon_purpur.. (+)	1	3,261	80.74	88.40	284
CepurGG1.1G272.. (+)	1	3,261	80.80	88.40	282
PpCESA3 (+)	1	3,282	80.58	88.97	311
Physcomitrium_pa.. (+)	1	3,282	80.58	88.97	311
PpCESA8 (+)	1	3,279	80.91	88.89	296
Physcomitrium_pa.. (+)	1	3,279	80.91	88.89	296
Foant17125 (+)	1	3,294	80.50	89.29	326
Fontinalis_antipyr.. (+)	1	3,294	80.39	89.29	330
Entjg12777 (+)	1	3,294	80.58	89.29	323
Entodon_seductrix_8 (+)	1	3,213	80.89	87.10	318
Hypjg10337 (+)	1	3,294	81.23	89.29	299
Hypnum_curvifoli.. (+)	1	3,242	79.86	87.88	296
Entodon_seductrix_3 (+)	1	3,294	80.82	89.29	314
Hypnum_curvifoli.. (+)	1	1,599	75.75	43.35	156
CepurR40.g02225 (+)	1	3,246	75.89	87.99	448
Sphfalx06G044300 (+)	1	3,276	80.29	88.80	318
Sphagnum_fallax_2 (+)	1	3,276	80.29	88.80	318
Sphmag06G057300 (+)	1	3,276	80.37	88.80	315
Sphagnum_magel.. (+)	1	3,276	80.37	88.80	315
Sphfalx09G034600 (+)	1	3,414	79.89	92.55	471
Sphagnum_fallax_1 (+)	1	3,414	79.89	92.55	471
Sphmag09G077200 (+)	1	3,411	79.59	92.46	479
Sphagnum_magel.. (+)	1	3,411	79.59	92.46	479
Mapoly0015s0055 (+)	1	3,297	76.28	89.37	487
Marchantia_polym.. (+)	1	3,297	76.28	89.37	487
Pm001908g0030 (+)	1	3,297	69.98	89.37	719
Pm001158g0050 (+)	1	3,300	69.90	89.46	725
Penium_margarita.. (+)	1	2,487	78.11	67.42	460
Penium_margarita.. (+)	1	1,878	77.36	50.91	344
Penium_margarita.. (+)	1	2,487	78.11	67.42	460
