# Supplementary material for: Diversification of Cellulose Synthase (CESA) Genes in Mosses Suggests Both Ancient and Recent Gene duplications
Source: J Mol Evol. 2026 Sep 16;94(5):907–21. doi: 10.1007/s00239-026-10342-3 (PMC13672439; doi:10.1007/s00239-026-10342-3)

NCBI Multiple Sequence Alignment Viewer, Version 1.26.0

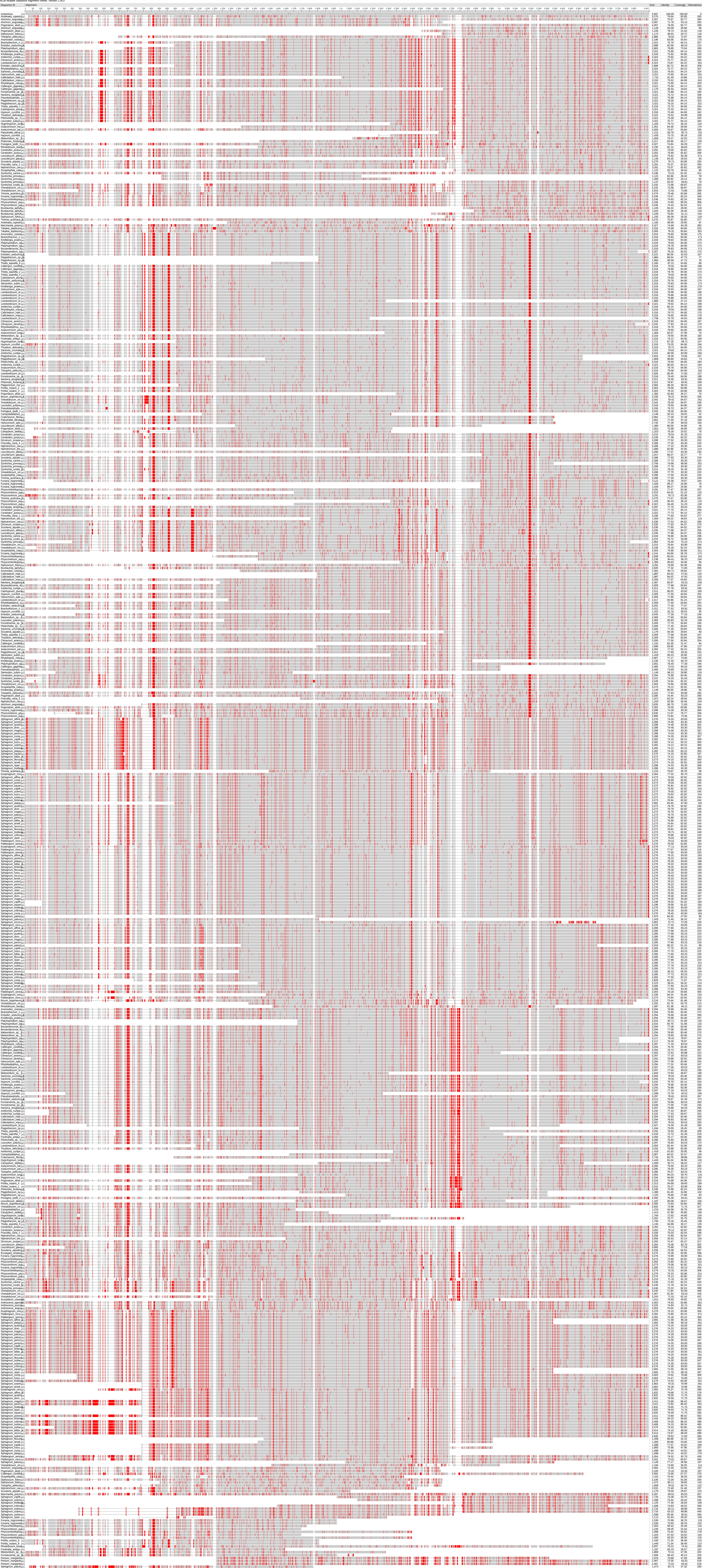

Supplement: Supplementary file 2 [file 239_2026_10342_MOESM2_ESM.pdf]
