# Supplementary material for: Diversification of Cellulose Synthase (CESA) Genes in Mosses Suggests Both Ancient and Recent Gene duplications
Source: J Mol Evol. 2026 Sep 16;94(5):907–21. doi: 10.1007/s00239-026-10342-3 (PMC13672439; doi:10.1007/s00239-026-10342-3)
Supplement: Supplementary file 3 [file 239_2026_10342_MOESM5_ESM.pdf]

Supplementary Table 1. Species used to examine the relationship among CESA genes, including taxonomic information, the number of genes identified in the assembled genomes, and the publication of the assembly.

**Class**	**Order**	**Family**	**Genus**	**Species**	**# genes**	**Reference**	**Assembly location**
Bryopsida	Dicranales	Ditrichaceae	*Ceratodon*	*purpureus* GG1	5	Carey et al., 2021	https://phytozome-next.jgi.doe.gov/
Bryopsida	Dicranales	Ditrichaceae	*Ceratodon*	*purpureus* R40	5	Carey et al., 2021	https://phytozome-next.jgi.doe.gov/
Bryopsida	Hypnales	Fontinalaceae	*Fontinalis*	*antipyretica*	4	Yu et al., 2020	http://gigadb.org/dataset/100748
Sphagnopsida	Sphagnales	Sphagnaceae	*Sphagnum*	*fallax*	6	Weston et al., 2018	https://phytozome-next.jgi.doe.gov/
Sphagnopsida	Sphagnales	Sphagnaceae	*Sphagnum*	*magellanicum*	6	Weston et al., 2018	https://phytozome-next.jgi.doe.gov/
Bryopsida	Hypnales	Entodontaceae	*Entodon*	*seductrix*	4	Yu et al., 2022	https://figshare.com/articles/dataset/Entodon_and_Hypnum_genome_project/17097077
Bryopsida	Hypnales	Hypnaceae	*Hypnum*	*curvifolium*	4	Yu et al., 2022	https://figshare.com/articles/dataset/Entodon_and_Hypnum_genome_project/17097077
Bryopsida	Funariales	Funariaceae	*Physcomitrium*	*patens*	8	Lang et al., 2018	https://phytozome-next.jgi.doe.gov/
Takakiopsida	Takakiales	Takakiaceae	*Takakia*	*lepidozioides*	2	Hu et al., 2023	https://www.takakia.com
Anthocerotopsida	Anthocerotales	Anthocerotaceae	*Anthoceros*	*punctatus*	1	Zhang et al., 2020	https://www.hornworts.uzh.ch/en/Blast.html
Anthocerotopsida	Anthocerotales	Anthocerotaceae	*Anthoceros*	*agrestis*	1	Zhang et al., 2020	https://www.hornworts.uzh.ch/en/Blast.html
Marchantiopsida	Marchatiales	Marchatiaceae	*Marchantia*	*polymorpha*	2	Bowman et al., 2017	https://marchantia.info/
Anthocerotopsida	Anthocerotales	Anthocerotaceae	*Anthoceros*	*angustus*	1	Zhang et al., 2020	https://www.hornworts.uzh.ch/en/Blast.html
Zygnematophyceae	Zygnematales	Zynemataceae	*Penium*	*margaritaceum*	3	Jiao et al., 2020	https://phytozome-next.jgi.doe.gov/

Supplementary Table 2: Species for which CESA genes were extracted from short-read sequence data. In cases where no genes could be assembled, sequencing depth might have been insufficient. Stars after gene numbers indicate that additional viable contigs (and therefore potential paralogs) were observed but were excluded due to short length.

**Species**	**Order**	**SRA/CNGD accession**	**Data type**	**Assembler**	**# Genes**
Andreaea regularis	Andreaeales	SRR22696218	WGS	Geneious	0
Andreaea rupestris	Andreaeales	ERR2040947	RNAseq	rnaSPAdes	2
Anthoceros agrestis	Anthocerotales	ERR3580402, ERR771108, ERR771109	WGS	Geneious	1
Anthoceros angustus	Anthocerotales	ERR9662965	RNAseq	rnaSPAdes	1
Anthoceros punctatus	Anthocerotales	ERR1278954	WGS	Geneious	1
Philonotis fontana	Bartramiales	ERR2040948	RNAseq	rnaSPAdes	3*
Bryum argenteum	Bryales	SRR1763242	RNAseq	rnaSPAdes	3
Plagiomnium insigne	Bryales	ERR2040949	RNAseq	rnaSPAdes	2
Plagiomnium rostratum	Bryales	SRR28576469	WGS	Geneious	0
Plagiomnium succulentum	Bryales	SRR28576968	WGS	Geneious	0
Plagiomnium vesicatum	Bryales	SRR28577168	WGS	Geneious	0
Pohlia nutans	Bryales	SRR494108	RNAseq	rnaSPAdes	6*
Rhodobryum laxelimbatum	Bryales	SRR14332107	WGS	Geneious	3*
Buxbaumia aphylla	Buxbaumiales	ERR2040954	RNAseq	rnaSPAdes	4
Penium margaritaceum	Desmidiales	SRR10442432	WGS	Geneious	3
Dicranum scoparium	Dicranales	SRR11912463	RNAseq	rnaSPAdes	3
Leucobryum albidum	Dicranales	ERR2040956	RNAseq	rnaSPAdes	6
Leucobryum glaucum	Dicranales	ERR2040957	RNAseq	rnaSPAdes	4
Diphyscium foliosum	Diphysciales	ERR2040958	RNAseq	rnaSPAdes	5
Encalypta streptocarpa	Encalyptales	ERR2040959	RNAseq	rnaSPAdes	2
Funaria hygrometrica	Funariales	ERR3958995	RNAseq	rnaSPAdes	10
Physcomitrellopsis africana	Funariales	SRR26586950	RNAseq	rnaSPAdes	9
Physcomitrium patens	Funariales	SRR3099021	WGS	Geneious	7
Physcomitrium pyriforme	Funariales	SRR10075982	RNAseq	rnaSPAdes	8
Frisvollia varia	Grimmiales	ERR2040952	RNAseq	rnaSPAdes	5
Niphotrichum canescens	Grimmiales	SRR14533271	RNAseq	rnaSPAdes	6
Niphotrichum elongatum	Grimmiales	ERR2040951	RNAseq	rnaSPAdes	3

Alleniella valentiniana	Hypnales	SRR17422315	WGS	Geneious	0
Amblystegium serpens	Hypnales	SRR26398974	WGS	Geneious	0
Anomodon rostratus	Hypnales	SRR2518087	RNAseq	rnaSPAdes	4*
Antitrichia curtipendula	Hypnales	SRR2518092	RNAseq	rnaSPAdes	9
Arvernella microclada	Hypnales	SRR26398973	WGS	Geneious	0
Brachythecium rivulare	Hypnales	SRR12006106, SRR12006107	RNAseq	rnaSPAdes	4
Bryoandersonia illecebra	Hypnales	SRR2518072	RNAseq	rnaSPAdes	5
Callicladium haldanianum	Hypnales	SRR2518077	RNAseq	rnaSPAdes	6
Callicladium imponens	Hypnales	SRR2513252	RNAseq	rnaSPAdes	4
Calliergon cordifolium	Hypnales	ERR2040964	RNAseq	rnaSPAdes	5
Calliergon giganteum	Hypnales	SRR2518083	RNAseq	rnaSPAdes	5*
Calohypnum plumiforme	Hypnales	SRR14748365	RNAseq	rnaSPAdes	4
Campyliadelphus chrysophyllus	Hypnales	SRR21785615, SRR26398951, SRR26398962	WGS	Geneious	3*
Campylium protensum	Hypnales	SRR21785614	WGS	Geneious	0
Campylium stellatum	Hypnales	SRR26398944, SRR26398945, SRR26398946	WGS	Geneious	3*
Campylophyllum halleri	Hypnales	SRR26398943	WGS	Geneious	0
Climacium americanum	Hypnales	SRR2497426	RNAseq	rnaSPAdes	4
Climacium dendroides	Hypnales	ERR2040965	RNAseq	rnaSPAdes	2
Cratoneuron curvicaule	Hypnales	SRR21785610	WGS	Geneious	0
Cratoneuron filicinum	Hypnales	SRR26398970	WGS	Geneious	2*
Cratoneuropsis relaxa	Hypnales	SRR21785607	WGS	Geneious	0
Drepanocladus aduncus	Hypnales	SRR26398966, SRR26398967, SRR26398968	WGS	Geneious	0
Drepanocladus polygamus	Hypnales	SRR26398964, SRR26398965	WGS	Geneious	0
Drepanocladus sordidus	Hypnales	SRR26398963	WGS	Geneious	0
Drepanocladus turgescens	Hypnales	SRR21785606	WGS	Geneious	0
Entodon seductrix	Hypnales	CL100124498	WGS	Geneious	8
Eurhynchiadelphus eustegia	Hypnales	SRR15179248	WGS	Geneious	0
Fontinalis antipyretica	Hypnales	ERR2040966	RNAseq	rnaSPAdes	3
Forsstroemia sp.	Hypnales	SRR2518099	RNAseq	rnaSPAdes	6
Hygrohypnella ochracea	Hypnales	SRR26398959, SRR26398960, SRR26398961	WGS	Geneious	0
Hygrohypnum luridum	Hypnales	SRR21785605, SRR26398953,	WGS	SPAdes	4*

		SRR26398955, SRR26398956, SRR26398957, SRR26398958			
Hylocomium splendens	Hypnales	SRR2518082	RNAseq	rnaSPAdes	5*
Hypnum curvifolium	Hypnales	CL100092064	WGS	SPAdes	7
Kandaea elodes	Hypnales	SRR21785604	WGS	Geneious	0
Kindbergia praelonga	Hypnales	SRR2518076	RNAseq	rnaSPAdes	7
Leptodictyum riparium	Hypnales	SRR21785603	WGS	Geneious	0
Leucodon julaceus	Hypnales	ERR2040963	RNAseq	rnaSPAdes	4
Loeskeobryum brevirostre	Hypnales	SRR2518080	RNAseq	rnaSPAdes	13*
Meteoridium sp.	Hypnales	SRR2518094	RNAseq	rnaSPAdes	7
Neckera douglasii	Hypnales	ERR2040967	RNAseq	rnaSPAdes	4
Palustriella falcata	Hypnales	SRR21785613, SRR26398952	WGS	SPAdes	3*
Pilotrichella sp.	Hypnales	SRR2518090	RNAseq	rnaSPAdes	4
Plagiothecium sp.	Hypnales	SRR2518105	RNAseq	rnaSPAdes	11
Planicladium nitidulum	Hypnales	SRR15264989	WGS	Geneious	0
Platyhypnidium sp.	Hypnales	SRR2518103	RNAseq	rnaSPAdes	9
Platyhypnum bestii	Hypnales	SRR26398950	WGS	Geneious	0
Platyhypnum smithii	Hypnales	SRR21785612	WGS	Geneious	0
Pseudotaxiphyllum elegans	Hypnales	ERR2040962	RNAseq	rnaSPAdes	4*
Rhytidiadelphus sp.	Hypnales	SRR2518073	RNAseq	rnaSPAdes	4
Rhytidiopsis robusta	Hypnales	SRR2518096	RNAseq	rnaSPAdes	4
Sanionia uncinata	Hypnales	SRR21369222, SRR21369242	RNAseq	rnaSPAdes	5
Sciaromiopsis sinensis	Hypnales	SRR15179250	WGS	Geneious	0
Scorpidium cossonii	Hypnales	SRR26398948, SRR26398949	WGS	Geneious	1*
Stereodon subimponens	Hypnales	ERR2040961	RNAseq	rnaSPAdes	4
Thelia asprella	Hypnales	SRR2518088	RNAseq	rnaSPAdes	8
Thuidium delicatulum	Hypnales	SRR2499574	RNAseq	rnaSPAdes	4
Tomentypnum nitens	Hypnales	SRR26398947	WGS	Geneious	0
Vittia pachyloma	Hypnales	SRR21785611	WGS	Geneious	0
Marchantia polymorpha	Marchantiales	SRR396657, SRR396658	WGS	Geneious	2
Pulvigera lyellii	Orthotrichales	ERR2040968	RNAseq	rnaSPAdes	3
Atrichum angustatum	Polytrichales	ERR2040969	RNAseq	rnaSPAdes	5
Pogonatum aloides	Polytrichales	SRR3737504	RNAseq	rnaSPAdes	8
Scopelophila cataractae	Pottiales	SRR17281053	RNAseq	rnaSPAdes	6
Syntrichia caninervis	Pottiales	SRR13777019	RNAseq	rnaSPAdes	4

Syntrichia princeps	Pottiales	ERR2040970	RNAseq	rnaSPAdes	7
Syntrichia ruralis	Pottiales	SRR27955585	WGS	SPAdes	5
Vinealobryum vineale	Pottiales	SRR24966267	RNAseq	rnaSPAdes	14
Ceratodon purpureus GG1	Pseudoditrichales	SRR1592642, SRR1592643, SRR3928188, SRR3928189, SRR3928190, SRR3928191, SRR3928192	WGS	Geneious	5
Ceratodon purpureus R40	Pseudoditrichales	SRR4125803, SRR4125804	WGS	Geneious	5
Aulacomnium heterostichum	Rhizogoniales	ERR2040950	RNAseq	rnaSPAdes	4
Aulacomnium palustre	Rhizogoniales	SRR2518059	RNAseq	rnaSPAdes	4
Aulacomnium turgidum	Rhizogoniales	SRR22904711, SRR22904712, SRR22904713	RNAseq	rnaSPAdes	2
Scouleria aquatica	Scouleriales	ERR2040953	RNAseq	rnaSPAdes	6
Eosphagnum inretortum	Sphagnales	ERR2324899	RNAseq	rnaSPAdes	5
Flatbergium novo-caledoniae	Sphagnales	ERR2324894	RNAseq	rnaSPAdes	6
Flatbergium sericeum	Sphagnales	ERR2324904	RNAseq	rnaSPAdes	5
Sphagnum affine	Sphagnales	SRR18184152	WGS	SPAdes	6
Sphagnum austinii	Sphagnales	SRR18184155	WGS	SPAdes	6
Sphagnum capillifolium	Sphagnales	ERR4778777, ERR4778814, ERR4778886	WGS	SPAdes	7*
Sphagnum compactum	Sphagnales	ERR4778615, ERR4780970, ERR4781616	WGS	SPAdes	6*
Sphagnum cribrosum	Sphagnales	ERR2324889	RNAseq	rnaSPAdes	6
Sphagnum divinum	Sphagnales	SRR18184161	WGS	SPAdes	6
Sphagnum fallax	Sphagnales	SRR4136564, SRR4136566	WGS	Geneious	6
Sphagnum fimbriatum	Sphagnales	ERR4778602, ERR4778784, ERR4778855	WGS	SPAdes	6
Sphagnum flexuosum	Sphagnales	ERR4778558, ERR4778564, ERR4778826	WGS	SPAdes	6*
Sphagnum fuscum	Sphagnales	ERR4778574, ERR4778864, ERR4780958	WGS	Geneious	6*
Sphagnum junghuhnianum	Sphagnales	SRR15858706	WGS	Geneious	0

Sphagnum lindbergii	Sphagnales	ERR4778812, ERR4780987, ERR4785889	WGS	Geneious	7
Sphagnum magellanicum	Sphagnales	SRR4996476, SRR4996477	WGS	Geneious	6
Sphagnum multifibrosum	Sphagnales	SRR15966551	WGS	Geneious	0
Sphagnum palustre	Sphagnales	ERR2040971	RNAseq	rnaSPAdes	8*
Sphagnum perichaetiale	Sphagnales	SRR18184153	WGS	SPAdes	6
Sphagnum platyphyllum	Sphagnales	ERR4778580, ERR4778597, ERR4778871	WGS	SPAdes	6*
Sphagnum portoricense	Sphagnales	SRR18427469	WGS	SPAdes	6
Sphagnum recurvum	Sphagnales	ERR2040972	RNAseq	rnaSPAdes	6*
Sphagnum riparium	Sphagnales	ERR4778610, ERR4778780, ERR4778850	WGS	SPAdes	7*
Sphagnum squarrosum	Sphagnales	ERR4778611	WGS	SPAdes	6*
Sphagnum subnitens	Sphagnales	ERR4778894, ERR4780988, ERR4781005	WGS	SPAdes	7
Sphagnum subsecundum	Sphagnales	ERR477405	RNAseq	rnaSPAdes	10
Sphagnum tenellum	Sphagnales	ERR4778604, ERR4778834, ERR4785892	WGS	Geneious	6
Takakia lepidozioides	Takakiales	SRR17573916, SRR17573917, SRR17573918, SRR17573919, SRR17573920	WGS	Geneious	2
Tetraphis pellucida	Tetraphidales	ERR2040974	RNAseq	rnaSPAdes	5
Timmia austriaca	Timmiales	ERR2040975	RNAseq	rnaSPAdes	5*

Supplementary Table 3: Lengths for CESA sequences recovered from genomic or transcriptomic Illumina data.

Name	Original contig no.	Sequence no.	Sequence length (bp)
Andreaea rupestris	1	1	3270
Andreaea rupestris	2	2	1182
Anomodon rostratus	1	1	3315
Anomodon rostratus	2	2	3294
Anomodon rostratus	3	3	2196
Anomodon rostratus	4	4	2361
Anthoceros agrestis	n.a.	1	3303
Anthoceros angustus	n.a.	1	3303
Anthoceros punctatus	n.a.	1	3225
Antitrichia curtipendula	1	1	3315
Antitrichia curtipendula	2	2	3324
Antitrichia curtipendula	3	3	3318
Antitrichia curtipendula	4	4	3321
Antitrichia curtipendula	5	5	3312
Antitrichia curtipendula	6	6	3192
Antitrichia curtipendula	7	7	3192
Antitrichia curtipendula	8	8	1419
Atrichum angustatum	1	1	3267
Atrichum angustatum	2	2	2835
Atrichum angustatum	3	3	2097
Atrichum angustatum	4	4	2094
Atrichum angustatum	5	5	1128
Aulacomnium heterostichum	1	1	3285
Aulacomnium heterostichum	2	2	3318
Aulacomnium heterostichum	3	3	2352
Aulacomnium heterostichum	4	4	1890
Aulacomnium palustre	1	1	3318
Aulacomnium palustre	2	2	3285
Aulacomnium palustre	3	3	3309
Aulacomnium palustre	4	4	3282
Aulacomnium turgidum	1	1	2001
Aulacomnium turgidum	3	2	1464
Brachythecium rivulare	1	1	3318
Brachythecium rivulare	2	2	3294

Brachythecium rivulare	3	3	3321
Brachythecium rivulare	4	4	3312
Bryoandersonia illecebra	1	1	3318
Bryoandersonia illecebra	2	2	3294
Bryoandersonia illecebra	4	3	3294
Bryoandersonia illecebra	5	4	3321
Bryoandersonia illecebra	6	5	3309
Bryum argenteum	1	1	3339
Bryum argenteum	2	2	3309
Bryum argenteum	3	3	3216
Buxbaumia aphylla	1	1	2826
Buxbaumia aphylla	2	2	1227
Buxbaumia aphylla	3	3	1104
Buxbaumia aphylla	4	4	1041
Callicladium haldanianum	1	1	3318
Callicladium haldanianum	2	2	3294
Callicladium haldanianum	3	3	3294
Callicladium haldanianum	4	4	1731
Callicladium haldanianum	5	5	1887
Callicladium haldanianum	6	6	1887
Callicladium imponens	1	1	3318
Callicladium imponens	2	2	3297
Callicladium imponens	3	3	3318
Callicladium imponens	4	4	3309
Calliergon cordifolium	1	1	3318
Calliergon cordifolium	2	2	3294
Calliergon cordifolium	3	3	2655
Calliergon cordifolium	4	4	2403
Calliergon cordifolium	5	5	1242
Calliergon giganteum	1	1	3321
Calliergon giganteum	2	2	3318
Calliergon giganteum	3	3	3294
Calliergon giganteum	4	4	2385
Calliergon giganteum	5	5	1170
Calohypnum plumiforme	1	1	3321
Calohypnum plumiforme	2	2	3318
Calohypnum plumiforme	3	3	3297
Calohypnum plumiforme	4	4	2511
Campyliadelphus chrysophyllus	1	1	1146

Campyliadelphus chrysophyllus	2	2	2067
Campyliadelphus chrysophyllus	3	3	1215
Campylium stellatum	1	1	1323
Campylium stellatum	2	2	1509
Campylium stellatum	4	3	1413
Ceratodon purpureus GG1	1	1	3294
Ceratodon purpureus GG1	2	2	3210
Ceratodon purpureus GG1	3	3	3321
Ceratodon purpureus GG1	4	4	3261
Ceratodon purpureus GG1	5	5	3261
Ceratodon purpureus R40	1	1	3222
Ceratodon purpureus R40	2	2	3321
Ceratodon purpureus R40	3	3	3249
Ceratodon purpureus R40	4	4	3261
Ceratodon purpureus R40	5	5	3261
Climacium americanum	1	1	3324
Climacium americanum	2	2	3318
Climacium americanum	3	3	3297
Climacium americanum	4	4	3309
Climacium dendroides	1	1	3261
Climacium dendroides	2	2	3242
Cratoneuron filicinum	1	1	2664
Cratoneuron filicinum	2	2	2718
Dicranum scoparium	1	1	3288
Dicranum scoparium	2	2	3330
Dicranum scoparium	3	3	3255
Diphyscium foliosum	1	1	3291
Diphyscium foliosum	2	2	2106
Diphyscium foliosum	3	3	1572
Diphyscium foliosum	4	4	1173
Diphyscium foliosum	5	5	1155
Encalypta streptocarpa	1	1	3297
Encalypta streptocarpa	2	2	3270
Entodon seductrix	1	1	3222
Entodon seductrix	2	2	3045
Entodon seductrix	3	3	3294
Entodon seductrix	4	4	2688
Entodon seductrix	5	5	3318
Entodon seductrix	6	6	3042

Entodon seductrix	7	7	2688
Entodon seductrix	8	8	3213
Eosphagnum inretortum	1	1	3264
Eosphagnum inretortum	3	2	3276
Eosphagnum inretortum	4	3	3279
Eosphagnum inretortum	5	4	2991
Eosphagnum inretortum	6	5	2190
Flatbergium novo-caledoniae	1	1	3276
Flatbergium novo-caledoniae	3	2	3282
Flatbergium novo-caledoniae	4	3	3276
Flatbergium novo-caledoniae	6	4	3342
Flatbergium novo-caledoniae	7	5	3273
Flatbergium novo-caledoniae	8	6	3276
Flatbergium sericeum	1	1	3276
Flatbergium sericeum	2	2	3282
Flatbergium sericeum	3	3	3270
Flatbergium sericeum	4	4	3303
Flatbergium sericeum	5	5	3264
Fontinalis antipyretica	1	1	3318
Fontinalis antipyretica	2	2	3294
Fontinalis antipyretica	3	3	1167
Forsstroemia sp.	1	1	3318
Forsstroemia sp.	3	2	3321
Forsstroemia sp.	4	3	3297
Forsstroemia sp.	5	4	3309
Forsstroemia sp.	6	5	3039
Forsstroemia sp.	8	6	1584
Frisvollia varia	1	1	3336
Frisvollia varia	2	2	3258
Frisvollia varia	3	3	3261
Frisvollia varia	4	4	3288
Frisvollia varia	5	5	1971
Funaria hygrometrica	1	1	3285
Funaria hygrometrica	2	2	3276
Funaria hygrometrica	4	3	3246
Funaria hygrometrica	5	4	3288
Funaria hygrometrica	6	5	3121
Funaria hygrometrica	7	6	2241
Funaria hygrometrica	9	7	1530

Funaria hygrometrica	10	8	1530
Funaria hygrometrica	11	9	1026
Funaria hygrometrica	12	10	1026
Hygrohypnum luridum	1	1	2712
Hygrohypnum luridum	2	2	1764
Hygrohypnum luridum	3	3	1425
Hygrohypnum luridum	4	4	1443
Hylocomium splendens	1	1	3321
Hylocomium splendens	2	2	3318
Hylocomium splendens	3	3	3294
Hylocomium splendens	4	4	3309
Hylocomium splendens	5	5	2745
Hypnum curvifolium	1	1	3309
Hypnum curvifolium	2	2	3318
Hypnum curvifolium	3	3	3318
Hypnum curvifolium	4	4	3242
Hypnum curvifolium	6	5	1428
Hypnum curvifolium	9	6	1422
Hypnum curvifolium	11	7	1599
Kindbergia praelonga	1	1	3318
Kindbergia praelonga	2	2	3318
Kindbergia praelonga	3	3	3294
Kindbergia praelonga	4	4	3294
Kindbergia praelonga	5	5	3318
Kindbergia praelonga	6	6	2238
Kindbergia praelonga	7	7	1140
Leucobryum albidum	1	1	3264
Leucobryum albidum	2	2	3288
Leucobryum albidum	3	3	3330
Leucobryum albidum	4	4	3252
Leucobryum albidum	5	5	1380
Leucobryum albidum	6	6	1167
Leucobryum glaucum	1	1	3330
Leucobryum glaucum	2	2	3060
Leucobryum glaucum	3	3	1146
Leucobryum glaucum	4	4	1017
Leucodon julaceus	1	1	3318
Leucodon julaceus	2	2	3297
Leucodon julaceus	3	3	3321

Leucodon julaceus	4	4	2460
Loeskeobryum brevirostre	1	1	3318
Loeskeobryum brevirostre	2	2	3318
Loeskeobryum brevirostre	3	3	3318
Loeskeobryum brevirostre	4	4	3324
Loeskeobryum brevirostre	5	5	3321
Loeskeobryum brevirostre	6	6	3297
Loeskeobryum brevirostre	8	7	3297
Loeskeobryum brevirostre	9	8	2826
Loeskeobryum brevirostre	10	9	2427
Loeskeobryum brevirostre	11	10	2417
Loeskeobryum brevirostre	12	11	1860
Loeskeobryum brevirostre	13	12	2055
Loeskeobryum brevirostre	14	13	1758
Marchantia polymorpha	1	1	3297
Marchantia polymorpha	2	2	3327
Meteoridium sp.	1	1	3318
Meteoridium sp.	2	2	3294
Meteoridium sp.	3	3	3294
Meteoridium sp.	4	4	3309
Meteoridium sp.	5	5	1659
Meteoridium sp.	6	6	1869
Meteoridium sp.	7	7	1842
Neckera douglasii	1	1	3321
Neckera douglasii	2	2	3297
Neckera douglasii	3	3	3312
Neckera douglasii	4	4	2487
Niphotrichum canescens	1	1	3336
Niphotrichum canescens	2	2	3258
Niphotrichum canescens	3	3	3288
Niphotrichum canescens	4	4	3261
Niphotrichum canescens	5	5	2031
Niphotrichum canescens	6	6	1203
Niphotrichum elongatum	1	1	2058
Niphotrichum elongatum	2	2	1920
Niphotrichum elongatum	3	3	1680
Palustriella falcata	1	1	2661
Palustriella falcata	2	2	2202
Palustriella falcata	4	3	1212

Penium margaritaceum	1	1	2487
Penium margaritaceum	2	2	2487
Penium margaritaceum	3	3	1878
Philonotis fontana	1	1	3312
Philonotis fontana	2	2	3246
Philonotis fontana	3	3	2217
Physcomitrellopsis africana	1	1	3273
Physcomitrellopsis africana	2	2	3291
Physcomitrellopsis africana	3	3	3279
Physcomitrellopsis africana	4	4	3246
Physcomitrellopsis africana	6	5	2508
Physcomitrellopsis africana	7	6	1998
Physcomitrellopsis africana	8	7	1353
Physcomitrellopsis africana	9	8	1365
Physcomitrellopsis africana	10	9	1500
Physcomitrium patens	1	1	3291
Physcomitrium patens	2	2	3282
Physcomitrium patens	3	3	3279
Physcomitrium patens	4	4	3168
Physcomitrium patens	5	5	2238
Physcomitrium patens	6	6	3030
Physcomitrium patens	7	7	1425
Physcomitrium pyriforme	1	1	3273
Physcomitrium pyriforme	2	2	3270
Physcomitrium pyriforme	3	3	3246
Physcomitrium pyriforme	4	4	2895
Physcomitrium pyriforme	5	5	2019
Physcomitrium pyriforme	6	6	2022
Physcomitrium pyriforme	7	7	1323
Physcomitrium pyriforme	8	8	1245
Pilotrichella sp.	1	1	3318
Pilotrichella sp.	2	2	3321
Pilotrichella sp.	3	3	3288
Pilotrichella sp.	4	4	3309
Plagiomnium insigne	1	1	2694
Plagiomnium insigne	2	2	2334
Plagiothecium sp.	1	1	3321
Plagiothecium sp.	2	2	3321
Plagiothecium sp.	3	3	3312

Plagiothecium sp.	4	4	2919
Plagiothecium sp.	5	5	1884
Plagiothecium sp.	6	6	1884
Plagiothecium sp.	7	7	1794
Plagiothecium sp.	8	8	1809
Plagiothecium sp.	11	9	1380
Plagiothecium sp.	13	10	1101
Plagiothecium sp.	14	11	1044
Platyhypnidium sp.	1	1	3318
Platyhypnidium sp.	2	2	3318
Platyhypnidium sp.	3	3	3257
Platyhypnidium sp.	4	4	2883
Platyhypnidium sp.	5	5	3117
Platyhypnidium sp.	6	6	3117
Platyhypnidium sp.	7	7	3051
Platyhypnidium sp.	8	8	2016
Platyhypnidium sp.	9	9	2016
Pogonatum aloides	1	1	3303
Pogonatum aloides	2	2	3267
Pogonatum aloides	3	3	3318
Pogonatum aloides	4	4	3285
Pogonatum aloides	5	5	2100
Pogonatum aloides	6	6	1851
Pogonatum aloides	7	7	1245
Pogonatum aloides	8	8	1392
Pohlia nutans	1	1	3315
Pohlia nutans	2	2	3303
Pohlia nutans	3	3	3303
Pohlia nutans	4	4	2313
Pohlia nutans	5	5	1440
Pohlia nutans	6	6	1440
Pseudotaxiphyllum elegans	1	1	3321
Pseudotaxiphyllum elegans	2	2	3330
Pseudotaxiphyllum elegans	3	3	3297
Pseudotaxiphyllum elegans	4	4	2409
Pulvigera lyellii	1	1	3318
Pulvigera lyellii	2	2	3300
Pulvigera lyellii	3	3	3327
Rhodobryum laxelimbatum	1	1	2937

Rhodobryum laxelimbatum	2	2	2235
Rhodobryum laxelimbatum	3	3	1587
Rhytidiadelphus sp.	1	1	3294
Rhytidiadelphus sp.	2	2	3318
Rhytidiadelphus sp.	3	3	3324
Rhytidiadelphus sp.	5	4	3309
Rhytidiopsis robusta	1	1	3318
Rhytidiopsis robusta	2	2	3321
Rhytidiopsis robusta	4	3	3297
Rhytidiopsis robusta	5	4	2907
Sanionia uncinata	1	1	3321
Sanionia uncinata	2	2	3321
Sanionia uncinata	3	3	3294
Sanionia uncinata	4	4	3294
Sanionia uncinata	5	5	3309
Scopelophila cataractae	1	1	3303
Scopelophila cataractae	3	2	3288
Scopelophila cataractae	4	3	3210
Scopelophila cataractae	5	4	3273
Scopelophila cataractae	7	5	1431
Scopelophila cataractae	8	6	1122
Scorpidium cossonii	n.a.	1	1611
Scouleria aquatica	1	1	3258
Scouleria aquatica	2	2	3288
Scouleria aquatica	3	3	3336
Scouleria aquatica	4	4	3279
Scouleria aquatica	5	5	1170
Scouleria aquatica	6	6	1179
Sphagnum affine	1	1	3285
Sphagnum affine	2	2	2832
Sphagnum affine	3	3	3276
Sphagnum affine	4	4	3273
Sphagnum affine	5	5	2691
Sphagnum affine	6	6	3276
Sphagnum austinii	1	1	3276
Sphagnum austinii	2	2	3276
Sphagnum austinii	3	3	3285
Sphagnum austinii	4	4	2832
Sphagnum austinii	5	5	3273

Sphagnum austinii	6	6	3288
Sphagnum capillifolium	1	1	3276
Sphagnum capillifolium	2	2	3285
Sphagnum capillifolium	3	3	3198
Sphagnum capillifolium	4	4	3273
Sphagnum capillifolium	5	5	3282
Sphagnum capillifolium	6	6	1698
Sphagnum capillifolium	7	7	1725
Sphagnum compactum	1	1	3276
Sphagnum compactum	2	2	3003
Sphagnum compactum	3	3	3273
Sphagnum compactum	4	4	3288
Sphagnum compactum	5	5	2829
Sphagnum compactum	6	6	1698
Sphagnum cribrosum	1	1	3273
Sphagnum cribrosum	2	2	3276
Sphagnum cribrosum	5	3	3276
Sphagnum cribrosum	6	4	3408
Sphagnum cribrosum	7	5	3282
Sphagnum cribrosum	8	6	1836
Sphagnum divinum	1	1	3276
Sphagnum divinum	2	2	2832
Sphagnum divinum	3	3	3276
Sphagnum divinum	4	4	3285
Sphagnum divinum	5	5	3273
Sphagnum divinum	6	6	3288
Sphagnum fallax	1	1	3414
Sphagnum fallax	2	2	3276
Sphagnum fallax	3	3	3276
Sphagnum fallax	4	4	3273
Sphagnum fallax	5	5	3285
Sphagnum fallax	6	6	3273
Sphagnum fimbriatum	1	1	3276
Sphagnum fimbriatum	2	2	3276
Sphagnum fimbriatum	3	3	3285
Sphagnum fimbriatum	4	4	3273
Sphagnum fimbriatum	5	5	3282
Sphagnum fimbriatum	6	6	2244
Sphagnum flexuosum	1	1	3276

Sphagnum flexuosum	2	2	3276
Sphagnum flexuosum	3	3	3285
Sphagnum flexuosum	4	4	3273
Sphagnum flexuosum	5	5	3273
Sphagnum flexuosum	6	6	1269
Sphagnum fuscum	1	1	3285
Sphagnum fuscum	2	2	3282
Sphagnum fuscum	3	3	3276
Sphagnum fuscum	4	4	3273
Sphagnum fuscum	5	5	3003
Sphagnum fuscum	6	6	1893
Sphagnum lindbergii	1	1	3273
Sphagnum lindbergii	2	2	3276
Sphagnum lindbergii	3	3	3288
Sphagnum lindbergii	4	4	3279
Sphagnum lindbergii	5	5	2832
Sphagnum lindbergii	6	6	2223
Sphagnum lindbergii	10	7	1125
Sphagnum magellanicum	1	1	3411
Sphagnum magellanicum	2	2	3276
Sphagnum magellanicum	3	3	3285
Sphagnum magellanicum	4	4	3276
Sphagnum magellanicum	5	5	3288
Sphagnum magellanicum	6	6	3273
Sphagnum palustre	1	1	3273
Sphagnum palustre	2	2	3276
Sphagnum palustre	3	3	3276
Sphagnum palustre	4	4	1875
Sphagnum palustre	5	5	2019
Sphagnum palustre	7	6	1425
Sphagnum palustre	8	7	1473
Sphagnum palustre	9	8	1128
Sphagnum perichaetiale	1	1	3411
Sphagnum perichaetiale	2	2	3276
Sphagnum perichaetiale	3	3	3276
Sphagnum perichaetiale	4	4	3285
Sphagnum perichaetiale	5	5	3273
Sphagnum perichaetiale	6	6	3288
Sphagnum platyphyllum	1	1	3285

Sphagnum platyphyllum	2	2	3276
Sphagnum platyphyllum	3	3	2691
Sphagnum platyphyllum	4	4	3282
Sphagnum platyphyllum	5	5	2682
Sphagnum platyphyllum	6	6	1698
Sphagnum portoricense	1	1	3411
Sphagnum portoricense	2	2	3276
Sphagnum portoricense	3	3	3276
Sphagnum portoricense	4	4	3285
Sphagnum portoricense	5	5	3273
Sphagnum portoricense	6	6	3288
Sphagnum recurvum	1	1	3414
Sphagnum recurvum	2	2	3276
Sphagnum recurvum	3	3	3276
Sphagnum recurvum	4	4	3273
Sphagnum recurvum	5	5	2853
Sphagnum recurvum	6	6	2340
Sphagnum riparium	1	1	3285
Sphagnum riparium	2	2	3276
Sphagnum riparium	3	3	2832
Sphagnum riparium	4	4	2691
Sphagnum riparium	5	5	3273
Sphagnum riparium	6	6	3288
Sphagnum riparium	7	7	1113
Sphagnum squarrosum	1	1	2691
Sphagnum squarrosum	2	2	2832
Sphagnum squarrosum	3	3	3285
Sphagnum squarrosum	4	4	3273
Sphagnum squarrosum	5	5	3198
Sphagnum squarrosum	6	6	3270
Sphagnum subnitens	1	1	2835
Sphagnum subnitens	2	2	3288
Sphagnum subnitens	3	3	3276
Sphagnum subnitens	4	4	3273
Sphagnum subnitens	5	5	3282
Sphagnum subnitens	6	6	2802
Sphagnum subnitens	7	7	1596
Sphagnum subsecundum	1	1	3285
Sphagnum subsecundum	2	2	3276

Sphagnum subsecundum	3	3	3276
Sphagnum subsecundum	4	4	3273
Sphagnum subsecundum	5	5	3276
Sphagnum subsecundum	9	6	3408
Sphagnum subsecundum	10	7	3270
Sphagnum subsecundum	14	8	2718
Sphagnum subsecundum	15	9	2718
Sphagnum subsecundum	16	10	1599
Sphagnum tenellum	1	1	3285
Sphagnum tenellum	2	2	3273
Sphagnum tenellum	3	3	3273
Sphagnum tenellum	4	4	3276
Sphagnum tenellum	5	5	2802
Sphagnum tenellum	6	6	1305
Stereodon subimponens	1	1	3318
Stereodon subimponens	2	2	3291
Stereodon subimponens	3	3	1419
Stereodon subimponens	4	4	1140
Syntrichia caninervis	1	1	3318
Syntrichia caninervis	2	2	3288
Syntrichia caninervis	3	3	3198
Syntrichia caninervis	5	4	3246
Syntrichia princeps	1	1	3288
Syntrichia princeps	2	2	3246
Syntrichia princeps	3	3	2964
Syntrichia princeps	4	4	2706
Syntrichia princeps	5	5	1308
Syntrichia princeps	6	6	1137
Syntrichia princeps	7	7	1122
Syntrichia ruralis	1	1	3315
Syntrichia ruralis	2	2	3249
Syntrichia ruralis	3	3	3213
Syntrichia ruralis	4	4	3192
Syntrichia ruralis	5	5	3246
Takakia lepidozioides	1	1	3309
Takakia lepidozioides	2	2	3318
Tetraphis pellucida	1	1	3315
Tetraphis pellucida	2	2	3285
Tetraphis pellucida	3	3	3318

Tetraphis pellucida	4	4	2880
Tetraphis pellucida	5	5	1608
Thelia asprella	1	1	3318
Thelia asprella	2	2	3318
Thelia asprella	3	3	3318
Thelia asprella	4	4	3291
Thelia asprella	5	5	3309
Thelia asprella	6	6	2169
Thelia asprella	7	7	2418
Thelia asprella	9	8	1191
Thuidium delicatulum	1	1	3315
Thuidium delicatulum	2	2	3318
Thuidium delicatulum	3	3	3291
Thuidium delicatulum	4	4	3309
Timmia austriaca	1	1	3288
Timmia austriaca	2	2	3270
Timmia austriaca	3	3	3279
Timmia austriaca	4	4	3279
Timmia austriaca	5	5	1215
Vinealobryum vineale	1	1	3246
Vinealobryum vineale	2	2	3240
Vinealobryum vineale	5	3	3315
Vinealobryum vineale	6	4	3288
Vinealobryum vineale	7	5	3243
Vinealobryum vineale	8	6	3192
Vinealobryum vineale	9	7	3342
Vinealobryum vineale	10	8	3303
Vinealobryum vineale	11	9	3342
Vinealobryum vineale	12	10	2916
Vinealobryum vineale	14	11	2487
Vinealobryum vineale	15	12	2832
Vinealobryum vineale	20	13	2202
Vinealobryum vineale	24	14	1194

Supplementary Table 4. Results of two-ratio branch model tests for variation in selective pressure among CESA lineages. Branches are ordered by false discovery rate (FDR)-adjusted significance. For each branch, the estimated foreground ω, likelihood ratio test statistic (2ΔlnL), p-value, FDR-adjusted p-value, and significance after FDR correction are shown.

Branch	ω	2ΔlnL	P	FDR	FDR Sig.
PpCESA10	0.5737	105.661	1.14e-23	5.46e-22	Yes
C_purpureus_GG1_v1_1_CepurGG1_7G024500_1_C.purpureus_GG1_v1.1_CepurGG1.7G024500.1	0.0155	23.711	7.10e-06	1.70e-04	Yes
Sc_g14392_V2_1A	0.0225	21.559	2.08e-05	3.33e-04	Yes
C_purpureus_GG1_v1_1_CepurGG1_6G157300_1_C.purpureus_GG1_v1.1_CepurGG1.6G157300.1	0.0229	19.349	6.29e-05	6.70e-04	Yes
Hyp_curivifolium_jg10340_t1	0.0001	19.140	6.98e-05	6.70e-04	Yes
Penium_pm001158g0050	0.0169	16.299	2.89e-04	2.31e-03	Yes
Eseductrix_jg12779_t1	0.0068	15.781	3.74e-04	2.57e-03	Yes
Penium_pm001908g0030	0.0148	12.841	1.63e-03	9.77e-03	Yes
Hyp_curivifolium_jg27744_t1	0.0147	11.966	2.52e-03	1.18e-02	Yes
PpCESA4	0.2228	11.658	2.94e-03	1.18e-02	Yes
S_fallax_v1_1_Sphfalx13G080300_1_S.fallax_v1.1_Sphfalx13G080300.1	0.5763	11.722	2.85e-03	1.18e-02	Yes
Sc_g11450_V2_1A	0.1176	11.778	2.77e-03	1.18e-02	Yes
PpCESA8	0.0274	10.596	5.00e-03	1.85e-02	Yes
PpCESA5	0.0402	9.457	8.84e-03	3.03e-02	Yes
PpCESA1	0.0951	8.898	1.17e-02	3.74e-02	Yes
AnthocerosagrBONN_evm_model_Sc2ySwM_228_2986_AnthocerosagrBONN_evm.model.Sc2ySwM_228	0.0241	7.574	2.27e-02	6.80e-02	No
S_magellanicum_v1_1_Sphmag13G079500_1_S.magellanicum_v1.1_Sphmag13G079500.1	0.3229	7.160	2.79e-02	7.87e-02	No
Foant_03732	0.0341	6.609	3.67e-02	9.79e-02	No
PpCESA3	0.0410	5.957	5.09e-02	1.29e-01	No
Sc_g02225_V2_1A	0.0464	5.549	6.24e-02	1.50e-01	No
Tle3c00983_1	0.0454	4.345	1.14e-01	2.60e-01	No
Eseductrix_jg6929_t1	0.0263	3.338	1.88e-01	4.11e-01	No
S_magellanicum_v1_1_Sphmag19G038600_1_S.magellanicum_v1.1_Sphmag19G038600.1	0.0185	3.171	2.05e-01	4.28e-01	No
Hyp_curivifolium_jg10337_t1	0.0349	2.881	2.37e-01	4.59e-01	No
Tle4c03737_1	0.0475	2.860	2.39e-01	4.59e-01	No
M_polymorpha_v3_Mapoly0076s0014_1_M.polymorpha_v3_Mapoly0076s0014.1_CDS	0.0508	2.430	2.97e-01	5.48e-01	No

S_fallax_v1_1_Sphfalx16G018300_1_S.fallax_v1.1_Sphfalx16G018300.1	0.1184	2.273	3.21e-01	5.66e-01	No
S_magellanicum_v1_1_Sphmag06G057300_1_S.magellanicum_v1.1_Sphmag06G057300.1	0.0272	2.217	3.30e-01	5.66e-01	No
PpCESA7	408.9630	2.078	3.54e-01	5.77e-01	No
S_fallax_v1_1_Sphfalx19G037000_1_S.fallax_v1.1_Sphfalx19G037000.1	0.0159	2.039	3.61e-01	5.77e-01	No
M_polymorpha_v3_1_Mapoly0015s0055_1_M.polymorpha_v3.1_Mapoly0015s0055.1_CDS	0.0772	1.622	4.44e-01	6.59e-01	No
S_magellanicum_v1_1_Sphmag09G077200_1_S.magellanicum_v1.1_Sphmag09G077200.1	0.1183	1.584	4.53e-01	6.59e-01	No
S_magellanicum_v1_1_Sphmag16G006100_1_S.magellanicum_v1.1_Sphmag16G006100.1	0.1287	1.704	4.27e-01	6.59e-01	No
Hyp_curivifolium_jg25231_t1	0.0958	1.156	5.61e-01	7.92e-01	No
AnthocerosANG012329_locus=scaffold76_len3303	0.0293	1.032	5.97e-01	7.94e-01	No
Eseductrix_jg16709_t1	0.0414	0.996	6.08e-01	7.94e-01	No
S_fallax_v1_1_Sphfalx06G044300_1_S.fallax_v1.1_Sphfalx06G044300.1	0.0333	0.982	6.12e-01	7.94e-01	No
Foant_17125	0.0504	0.865	6.49e-01	8.19e-01	No
C_purpureus_GG1_v1_1_CepurGG1_1G272400_1_C.purpureus_GG1_v1.1_CepurGG1.1G272400.1	0.0557	0.500	7.79e-01	9.34e-01	No
Eseductrix_jg12777_t1	0.0513	0.455	7.97e-01	9.34e-01	No
Foant_15610	0.0525	0.356	8.37e-01	9.34e-01	No
S_fallax_v1_1_Sphfalx09G034600_1_S.fallax_v1.1_Sphfalx09G034600.1	0.0434	0.361	8.35e-01	9.34e-01	No
S_fallax_v1_1_Sphfalx17G024100_1_S.fallax_v1.1_Sphfalx17G024100.1	0.0445	0.384	8.25e-01	9.34e-01	No
C_purpureus_GG1_v1_1_CepurGG1_1G272300_1_C.purpureus_GG1_v1.1_CepurGG1.1G272300.1	0.0598	0.193	9.08e-01	9.69e-01	No
Foant_02392	0.0705	0.192	9.08e-01	9.69e-01	No
C_purpureus_GG1_v1_1_CepurGG1_10G096400_1_C.purpureus_GG1_v1.1_CepurGG1.10G096400.1	0.0670	0.012	9.94e-01	9.98e-01	No
PpCESA6	0.0687	0.004	9.98e-01	9.98e-01	No
S_magellanicum_v1_1_Sphmag17G023300_1_S.magellanicum_v1.1_Sphmag17G023300.1	0.0667	0.004	9.98e-01	9.98e-01	No

Supplementary Table 5. Results of branch-site Model A tests for episodic positive selection across 48 foreground branches in the CESA phylogeny. Likelihood ratio tests compared the branch-site model to the corresponding null model with ω_2 fixed at 1, and p-values were adjusted using the Benjamini-Hochberg false discovery rate (FDR) procedure. Three foreground branches remained significant after FDR correction. No codons were identified as positively selected by Bayes Empirical Bayes analysis (posterior probability ≥ 0.95).

**Branch**	**ω2**	**2ΔlnL**	**P**	**FDR**	**FDR Sig.**	**Selected Sites (%)**	**BEB ≥ 0.95**
Foant_02392	1.5	54.208	1.80e-13	8.66e-12	Yes	4.24	0
C_purpureus_GG1_v1_1_CepurGG1_1G272300_1_C.purpureus_GG1_v1.1_CepurGG1.1G272300.1	1.5	13.783	2.05e-04	4.93e-03	Yes	1.52	0
M_polymorpha_v3_Mapoly0076s0014_1_M.polymorpha_v3_Mapoly0076s0014.1_CDS	1.5	12.102	5.04e-04	8.06e-03	Yes	1.59	0
M_polymorpha_v3_1_Mapoly0015s0055_1_M.polymorpha_v3.1_Mapoly0015s0055.1_CDS	1.5	6.846	8.88e-03	8.53e-02	No	4.37	0
Tle4c03737_1	1.5	7.067	7.85e-03	8.53e-02	No	0.91	0
C_purpureus_GG1_v1_1_CepurGG1_1G272400_1_C.purpureus_GG1_v1.1_CepurGG1.1G272400.1	1.5	5.536	1.86e-02	1.49e-01	No	0.99	0
Sc_g02225_V2_1A	1.5	3.598	5.79e-02	3.97e-01	No	1.59	0
PpCESA6	1.5	3.262	7.09e-02	4.25e-01	No	0.00	0
Foant_17125	1.5	1.842	1.75e-01	9.32e-01	No	0.76	0
AnthocerosagrBONN_evm_model_Sc2ySwM_228_2986_AnthocerosagrBONN_evm.model.Sc2ySwM_228	1.5	0.000	1.00e+00	1.00e+00	No	0.00	0
scaffold76_len3303	1.5	0.000	9.96e-01	1.00e+00	No	0.00	0
C_purpureus_GG1_v1_1_CepurGG1_10G096400_1_C.purpureus_GG1_v1.1_CepurGG1.10G096400.1	1.5	0.000	1.00e+00	1.00e+00	No	0.00	0
C_purpureus_GG1_v1_1_CepurGG1_6G157300_1_C.purpureus_GG1_v1.1_CepurGG1.6G157300.1	1.5	0.000	1.00e+00	1.00e+00	No	0.00	0
C_purpureus_GG1_v1_1_CepurGG1_7G024500_1_C.purpureus_GG1_v1.1_CepurGG1.7G024500.1	1.5	0.000	1.00e+00	1.00e+00	No	0.00	0
Eseductrix_jg12777_t1	1.5	0.000	1.00e+00	1.00e+00	No	0.00	0
Eseductrix_jg12779_t1	1.5	0.000	1.00e+00	1.00e+00	No	0.00	0
Eseductrix_jg16709_t1	1.5	0.000	9.99e-01	1.00e+00	No	0.00	0
Eseductrix_jg6929_t1	1.5	0.000	1.00e+00	1.00e+00	No	0.00	0
Foant_03732	1.5	0.000	1.00e+00	1.00e+00	No	0.00	0
Foant_15610	1.5	0.000	9.99e-01	1.00e+00	No	0.00	0

Branch	ω2	2ΔlnL	P	FDR	FDR Sig.	Selected Sites (%)	BEB ≥ 0.95
Hyp_curivifolium_jg10337_t1	1.5	0.000	1.00e+00	1.00e+00	No	0.00	0
Hyp_curivifolium_jg10340_t1	1.5	0.000	1.00e+00	1.00e+00	No	0.00	0
Hyp_curivifolium_jg25231_t1	1.5	-0.032	1.00e+00	1.00e+00	No	0.42	0
Hyp_curivifolium_jg27744_t1	1.5	0.000	9.99e-01	1.00e+00	No	0.00	0
Penium_pm001158g0050	1.5	0.000	1.00e+00	1.00e+00	No	0.00	0
Penium_pm001908g0030	1.5	0.259	6.11e-01	1.00e+00	No	0.29	0
PpCESA1	1.5	-4.993	1.00e+00	1.00e+00	No	0.00	0
PpCESA10	1.5	-9.469	1.00e+00	1.00e+00	No	100.00	0
PpCESA3	1.5	0.000	1.00e+00	1.00e+00	No	0.00	0
PpCESA4	1.5	0.397	5.29e-01	1.00e+00	No	5.69	0
PpCESA5	1.5	1.445	2.29e-01	1.00e+00	No	1.15	0
PpCESA7	1.5	-0.071	1.00e+00	1.00e+00	No	100.00	0
PpCESA8	1.5	0.000	1.00e+00	1.00e+00	No	0.00	0
Sc_g11450_V2_1A	1.5	0.000	9.96e-01	1.00e+00	No	0.00	0
Sc_g14392_V2_1A	1.5	0.000	1.00e+00	1.00e+00	No	0.00	0
S_fallax_v1_1_Sphfalx06G044300_1_S.fallax_v1.1_Sphfalx06G044300.1	1.5	0.000	1.00e+00	1.00e+00	No	0.00	0
S_fallax_v1_1_Sphfalx09G034600_1_S.fallax_v1.1_Sphfalx09G034600.1	1.5	0.000	9.99e-01	1.00e+00	No	0.00	0
S_fallax_v1_1_Sphfalx13G080300_1_S.fallax_v1.1_Sphfalx13G080300.1	1.5	-0.052	1.00e+00	1.00e+00	No	7.91	0
S_fallax_v1_1_Sphfalx16G018300_1_S.fallax_v1.1_Sphfalx16G018300.1	1.5	-0.444	1.00e+00	1.00e+00	No	6.86	0
S_fallax_v1_1_Sphfalx17G024100_1_S.fallax_v1.1_Sphfalx17G024100.1	1.5	-0.058	1.00e+00	1.00e+00	No	0.89	0
S_fallax_v1_1_Sphfalx19G037000_1_S.fallax_v1.1_Sphfalx19G037000.1	1.5	0.000	1.00e+00	1.00e+00	No	0.00	0
S_magellanicum_v1_1_Sphmag06G057300_1_S.magellanicum_v1.1_Sphmag06G057300.1	1.5	0.000	1.00e+00	1.00e+00	No	0.00	0
S_magellanicum_v1_1_Sphmag09G077200_1_S.magellanicum_v1.1_Sphmag09G077200.1	1.5	-0.157	1.00e+00	1.00e+00	No	2.08	0
S_magellanicum_v1_1_Sphmag13G079500_1_S.magellanicum_v1.1_Sphmag13G079500.1	1.5	-1.212	1.00e+00	1.00e+00	No	0.00	0
S_magellanicum_v1_1_Sphmag16G006100_1_S.magellanicum_v1.1_Sphmag16G006100.1	1.5	-0.107	1.00e+00	1.00e+00	No	3.29	0
S_magellanicum_v1_1_Sphmag17G023300_1_S.magellanicum_v1.1_Sphmag17G023300.1	1.5	1.109	2.92e-01	1.00e+00	No	1.28	0
S_magellanicum_v1_1_Sphmag19G038600_1_S.magellanicum_v1.1_Sphmag19G038600.1	1.5	0.000	1.00e+00	1.00e+00	No	0.00	0
Tle3c00983_1	1.5	-0.004	1.00e+00	1.00e+00	No	0.00	0

Supplementary Figure 1: The alignment of CESA genes identified by pBLAST with the PpCESA5 protein sequence from whole genome assemblies (Supplementary Table 1), combined with CESA genes recovered from genomic or transcriptomic Illumina data (Supplementary Tables 2 and 3) for the same species. Gene sequences were codon-aligned with the Translation Align tool in Geneious Prime using MAFFT as the aligner and checked by eye. Poorly aligned regions at the start and end of incomplete CESA contigs that resulted in large gaps were trimmed. The alignment was visualized using the NLM NCBI MSA Viewer 1.26.0. Gray bars indicate consistency with the consensus, while red lines indicate differences.

Supplementary Figure 2: The alignment of CESA genes recovered from genomic or transcriptomic Illumina data (Supplementary Tables 2 and 3). Gene sequences were codon-aligned with the Translation Align tool in Geneious Prime using MAFFT as the aligner and checked by eye. Poorly aligned regions at the start and end of incomplete CESA contigs that resulted in large gaps were trimmed. The alignment was visualized using the NLM NCBI MSA Viewer 1.26.0. Gray bars indicate consistency with the consensus, while red lines indicate differences.

Supplementary Figure 3: The complete phylogeny of CESA genes from all 130 species of mosses. Sequences were obtained by mapping short-read sequences against reference CESA gene sequences and assembling de novo. Nodes with bootstrap > 75 are marked with a grey circle.

Supplementary Figure 4. The phylogeny of CESA genes from 130 species of mosses including only nearly complete sequences in the phylogenetic reconstruction. Sequences were obtained by mapping short-read sequences against reference CESA gene sequences and assembling de novo. Some supported clades are collapsed to allow improved viewing. These are marked with a diamond and include Sphagnales and Hypnales. Nodes with bootstrap > 75 are marked with a grey circle.
